# Honey and Its Biomimetic Deep Eutectic Solvent Modulate the Antioxidant Activity of Polyphenols

**DOI:** 10.3390/antiox11112194

**Published:** 2022-11-06

**Authors:** Luminița Dimitriu, Diana Constantinescu-Aruxandei, Daniel Preda, Andra-Lavinia Nichițean, Cristian-Andi Nicolae, Victor Alexandru Faraon, Marius Ghiurea, Mihaela Ganciarov, Narcisa Elena Băbeanu, Florin Oancea

**Affiliations:** 1 Bioproducts Team, Bioresources Department, National Institute for Research & Development in Chemistry and Petrochemistry—ICECHIM, Splaiul Independenței No. 202, Sector 6, 060021 Bucharest, Romania; 2 Faculty of Biotechnologies, University of Agronomic Sciences and Veterinary Medicine of Bucharest, Mărăști Blv., No. 59, Sector 1, 011464 Bucharest, Romania; 3 Department of Analytical Chemistry and Environmental Engineering, Faculty of Chemical Engineering and Biotechnologies, University Politehnica Bucharest, Str. Gheorghe Polizu nr/1-7, Sector 1, 011061 Bucharest, Romania; 4 Research and Development Department, Rom Honey Group Srl, Str. Grădinari nr. 1, Iași County, 700390 Iași, Romania

**Keywords:** honey, natural deep eutectic solvent (NaDES), biomimetic NaDES, antioxidant activity, polyphenols, synergism, antagonism, raspberry extract, polyphenol-enriched honey

## Abstract

Honey is a highly valued natural product with antioxidant, antimicrobial and anti-inflammatory properties. However, its antioxidant activity (AOA) is not as high as that of other honeybee products, such as propolis. Several polyphenol—honey formulations have been proposed up to now, most of them using maceration of biomass in honey or mixtures with liquid extracts, which either limit polyphenols bioavailability or destroy the characteristics of honey. To improve the health benefits of honey by increasing AOA and keeping its structural and sensory properties, we propose its enrichment in a polyphenol extract of raspberry after solvent evaporation. A honey-biomimetic natural deep eutectic solvent (NaDES) was prepared and compared with honey. The main polyphenols found in the raspberry extract were tested in combination with honey and NaDES, respectively. The AOA was determined by DPPH, ABTS, CUPRAC, and FRAP methods. The AOA behaviour of honey—polyphenol mixtures varied from synergism to antagonism, being influenced by the AOA method, polyphenol type, and/or mixture concentration. The honey-biomimetic NaDES resulted in similar AOA behaviour as with honey mixed with polyphenols. Honey seems to have additional properties that increase synergism or reduce antagonism in some cases. Honey and its biomimetic NaDES modulate AOA of polyphenols extract.

## 1. Introduction

Honey is a natural product produced by honeybees with several biological properties resulting from its multifaceted activities, i.e., antioxidant, antimicrobial, and anti-inflammatory activities. The chemical composition depends on diverse factors such as sugary source, floral nectar or aphid honeydew, environmental conditions, and genetic factors; it consists of more than 80% sugar [1]. Honey is considered to have the characteristics of a natural deep eutectic solvent (NaDES) due to the intermolecular interactions between monosaccharides and disaccharides and hydrogen bonds formed between them [2]. Deep eutectic solvents (DES) are a class of solvents produced by mixing a minimum of two components that at ambient conditions remain together in a liquid state. The DES components have a melting point above that of the eutectic point due to hydrogen bonds that are formed between these components [3].

Polyphenols are secondary metabolites of plants with various biological activities, such as antioxidant, antimicrobial, prebiotic, enzyme inhibition activity, and others [4]. The AOA activity of honey is believed to be mainly the result of the presence of different categories of polyphenols. Compared with other types of honeybee products, such as propolis, honey has a lower AOA per gram of sample. Because honey remains one of the most consumed honeybee products, it is desirable to improve its health benefits, including its antioxidant capacity. One solution is to enrich honey in polyphenols from other sources.

Several previous studies have used different types of approaches, from maceration with unprocessed biomass rich in polyphenols such as propolis, beebread, royal jelly, pollen, plant leaves, and fruits, to mixtures with polyphenol extractions in different ratios [5,6,7,8,9,10]. In general, the mixtures have been prepared with liquid hydroalcoholic extraction, which does not preserve the characteristics and properties of honey during formulation. On the other hand, macerations in honey could have the disadvantage of limiting the bioavailability of compounds released from the biomass. Separating the unsolubilized residues from polyphenol-rich biomass is difficult due to the high viscosity of honey and restriction on honey heating, which increases the formation of 5-hydroxymethylfurfural (5-HMF) by dehydration of glucose or fructose [11]. Another issue related to honey enrichment with polyphenol-rich biomass (such as propolis) is deterioration of the sensory properties due to the astringency and bitterness of the polyphenols [12].

Raspberry (*Rubus idaeus*) fruits are a good source of polyphenols (phenolic acids, flavonoids, and anthocyanins) for human nutrition, with antioxidant properties and excellent sensory characteristics [13]. Raspberry leaves and fruits added to rape honey in amounts of 0.5% and 1% and, respectively, 1% and 4%, were already demonstrated to enhance the antioxidant properties of honey and to increase its antibacterial and antiviral characteristics [10].

In this study, we enriched honey with dry extracts of raspberry as an example of enriching honey in antioxidant polyphenols after evaporation of the solvent. In this way, the water activity, as well as structural features of honey during formulation, can be preserved and, therefore, its stability and properties. The resulting product is a honeybee product fortified with polyphenols, which has superior sensory characteristics compared with other possible combinations, such as honey and propolis, the latter having strong astringency properties. We also investigated the modulation potential between honey and polyphenols and exploited the contribution of the main sugars in a honey-mimicking DES formulation, sugars that are believed to give the main characteristics of honey as a natural DES.

## 2. Materials and Methods

### 2.1. Materials

Fresh raspberries (*R. idaeus*, cv. Remontant, from Domeniul Cerbi, Marginea, Suceava, Romania) and multifloral honey (RomHoney Group, Iași, Romania) were used in this work. The raspberries were dried by lyophilization and were ground to a fine powder using an electrical grinder. The following chemicals were used: pharmaceutical ethanol 96% (Chimopar Srl, Bucharest, Romania), D(+)-Glucose anhydrous extra pure, D(−)-Fructose, extra pure, D(+) Saccharose, reagent grade (Scharlau, Barcelona, Spain) Trolox 97% (Acros Organics, Thermo Fisher Scientific, Pittburghs, PA, USA), Gallic acid, 2,2-Diphenyl-1-picrylhydrazyl (Sigma-Aldrich, Merck Group, Darmstad, Germany), 2,2′-Azino-bis (3-ethylbenzothiazo-line-6-sulfonic acid) diammonium salt, 98%, 2,4,6-tri (2-pyridyl-1,3,5-triazine) 98% (Alfa Aesar, Kandel, Germany), Folin Ciocalteu’s phenol reagent, Iron chloride (III) (Merck, Darmstadt, Germany), hydrochloric acid, acetic acid (Chimopar Srl, Bucharest, Romania), sodium acetate (Scharlau, Barcelona, Spain), HPLC standards: ferulic acid, p-coumaric acid, caffeic acid, quercetin dihydrate (Sigma-Aldrich, Merck Group, Darmstad, Germany), syringic acid, luteolin, (+)-rutin trihydrate, (Alfa Aesar, Haverhill, MA, USA), chlorogenic acid, myricetin (Cayman Chemical, Ann Arbor, MI, USA), apigenin, (−) epicatechin (Roth, Karlsruhe, Germany), and kaempferol (Cayman Chemical, Ann Arbor, MI, USA).

### 2.2. Hydroalcoholic Extraction of Polyphenols from Raspberry

The polyphenols were extracted from freeze-dried raspberries by ultrasound-assisted extraction with a 70% ethanol solution, and a ratio of substrate to solvent of 1:10, for 30 min. at room temperature. The extraction was performed in an ultrasonic bath (P = 580 W, frequency = 37 Hz), with the temperature at 20–30 °C by adding ice to the bath. The samples were then centrifuged for 20 min at 8500 rpm, the supernatant was removed, and the same volume of solvent was added over the remaining substrate to repeat the extraction. The two resulting extract fractions were mixed together.

### 2.3. Analysis of Polyphenolic Content of Raspberry Extract and Honey

#### 2.3.1. HPLC Analysis

High-pressure liquid chromatographic (HPLC) analysis of phenolic acids and flavonoids was performed using Dionex Ultimate 3000 equipment (Thermo Fisher Scientific, Waltham, MA, USA) with VWD-3100 detector, and the chromatograms were processed by Chromelleon 7.0 software (Thermo Fisher Scientific, Waltham).

##### Solid-Phase Extraction

For the extraction of polyphenols from honey, solid-phase extraction was performed based on a previously described method [14] with some modifications. Five grams of honey were dissolved with 10 mL of MilliQ water and passed through a Strata^®^SDB-L-conditioned cartridge (100 µm styrene-divinylbenzene 500 mg/3 mL, Phenomenex, Torrance, CA, USA) with a mixture of acetonitrile, methanol, and MilliQ water (1:1:1), at a flow rate of 1 mL/min. Elution was effected with a mixture of Methanol-Acetonitrile 2:1 at 1 mL/min.

##### HPLC Analysis of Phenolic Acids

The analysis of phenolic acids was conducted according to a method described by [15] on a Luna Omega 5 μm Polar C18 100 Å column (250 mm × 4.6 mm) (Phenomenex, Torrance, CA, USA). The method involved using a gradient program with a two-solvent system (A: aqueous solution with 0.1% formic acid and B: methanol), applied as follows: 0–25 min. 5% B, 25–33 min. 30% B, 34–40 min. 5% B. The flow rate was set at 1.25 mL × min^−1,^ and an injection volume of 10 μL was used to detect phenolic acids at 280 nm. The calibration curve consisted of several standard concentrations between 18.125–1000 µg/mL. The coefficients of determination (R^2^) were above 0.9996, which indicated good linearity.

##### HPLC Analysis of Flavonoids

The HPLC analysis of flavonoids from the raspberry extract and honey was performed according to the method described by [16] on an Omega 5 μm Polar C18 100 Å column (250 mm × 4.6 mm) (Phenomenex, Torrance). The compounds were separated with a gradient elution of the mobile phase composed of (A) MeOH and (B) 0.5% H_3_PO_4_. The gradient elution program was set as follows: 0–10 min 15% A and 85% B, 15–25 min 85% A and 15% B, 25–30 min. 60% A and 40% B. The flow rate of the mobile phase was 1.5 mL/min, and the column temperature was 25 °C. to detect flavonoids at 280 nm.

Flavonoids were identified and quantified by matching the retention time and their spectral characteristics with the standards using a calibration curve.

#### 2.3.2. Total Polyphenol Content

The total polyphenol content (TPC) of the extracts was determined by the Folin-Ciocalteau method described by [17]. Briefly, 10 µL of sample solution or standard solution was mixed with 90 µL double-distilled water (ddH_2_O) and 10 µL of Folin Ciocalteu reagent. After 5 min of mixing, 100 µL of 7% Na_2_CO_3_ and 40 µL ddH_2_O were added to the mixture. The absorbance was measured spectrophotometrically using a plate reader (CLARIOstar, BMG LABTECH, Ortenberg, Germany) at 765 nm after 60 min of incubation at room temperature. The calibration curve was in the range of 5–30 µg/mL of gallic acid in 70% ethanol. The results were expressed as mg gallic acid equivalent/100 g dry weight (DW) of the sample (mg GAE/100 g).

#### 2.3.3. Total Flavonoid Content

The total flavonoid content (TFC) of the extracts was determined using the aluminum chloride/sodium acetate method according to [18] with some modifications. To evaluate the TFC, 0.1 mL of sample/standard was mixed with 0.1 mL of 10% sodium acetate and then 0.12 mL of 2.5% AlCl_3_ and 0.68 mL of ddH_2_O were added to the mixture. The absorbance was read at λ = 430 nm after 45 min of incubation at room temperature. The results were expressed as quercetin equivalent mg/100 g DW of the sample.

#### 2.3.4. Total Hydroxycinnamic Acid Content

Total hydroxycinnamic acid content (HAT) was determined by a method adapted from the European Pharmacopoeia [19]. Briefly, 0.25 µL of sample/standard was mixed with 50 µL 0.5 M HCl, then 50 µL of solution consisting of 1% (*w*/*v*) NaNO_2_ and 1% (*w*/*v*) Na_2_MoO_4_ were added, followed by 50 µL of 8.5% NaOH and 75 µL ddH_2_O. The absorbance was read at λ = 524 nm. A calibration curve with chlorogenic acid at concentrations in the range 0–50 µg/mL in 70% (*v*/*v*) ethanol was performed to quantify hydroxycinnamic acids. The results were expressed as mg chlorogenic acid equivalent/100 g DW of the sample.

#### 2.3.5. Total Anthocyanin Content

Total anthocyanin content (TAC) was determined by the pH differential spectroscopic method [20]. Briefly, 1.5 mL of extracts were diluted in two different buffers: in 0.025 M potassium chloride buffer pH = 1, and in 0.4 M sodium acetate buffer pH = 4.5 respectively. The absorbance (A) was measured at 520 and 700 nm (Ocean Optics UV-VIS-NIR, Orlando, FL, USA) after 30 min of incubation at room temperature. The TAC was calculated using the molar absorptivity coefficient (ε) and molecular weight (MW) of cyaniding 3-glucoside (ε = 26,900 M^−1^ cm^−1^ and MW = 449.2 g/mol). The results were calculated as follows: A_sp_ = (A_520_−A_700_)_pH1.0_−(A_520_−A_700_)_pH4.5_ and TAC = (A_sp_ × MW × DF × V × 1000)/(ε × L × m), where A_sp_ is the absorbance of sample, DF is dilution factor, L is the cuvette optical pathlength (1 cm), *V*-volume of the extracts (L), and m is the weight of the sample (g). TAC was expressed as mg cyaniding 3-glucoside equivalent/100 g DW of the sample.

### 2.4. Preparation of Honey with Raspberry Extract/Polyphenolic Standard for AOA Activities

The extracts of raspberry were split equally into two equal fractions that were concentrated to dryness (S_CD) using a semi-automated evaporation system, i.e., a MultiVap54 (Lab tech, Sorisole, Italy) at 40 °C. One of the fraction S_CD was resuspended in honey (H) at a ratio of 1:20 (*w*/*w*), resulting the honey-raspberry mixture sample (H_RE). The other fraction S_CD was resuspended in 70% ethanol solution at the same ratio as in honey (1:20 *w*/*v*), resulting in the RE sample. The sample H_RE was obtained by solubilizing the extract fraction S_CD in honey using an ultrasonic bath, mixing thoroughly, and leaving the polyphenols to diffuse overnight in honey. For AOA, the samples H and H_RE were solubilized in 70% (*v*/*v*) ethanol at a concentration of 0.2 g/mL (*w*/*v*). The AOA of the samples was assayed using four spectrophotometric methods: radical scavenging activity (ABTS and DPPH) and reducing antioxidant power (CUPRAC and FRAP). The AOA was performed at several concentrations, and calibration curves were calculated for each method. The concentration values of RE tested individually were equivalent to the concentrations of RE in mixtures with honey/GFSw. To check if the behaviour of RE held for individual polyphenols dissolved in honey, we prepared the mixture of honey and individual major polyphenols found in the raspberry extract: caffeic acid (CA) and epicatechin (EP). Each polyphenol was solubilized in honey/70% ethanol at 0.5 mg per g (*w*/*w*) of honey or 0.5 mg per mL (*w*/*v)* of 70% ethanol using an ultrasonic bath and the polyphenols were left to diffuse overnight. The AOA was performed at several concentrations, and calibration curves were calculated for each method, as in the case of RE. The concentration values of CA/EP tested individually were equivalent to the concentrations used in mixtures with honey/GFSw. The final concentrations tested were in the range of 5 to 200 mg/mL honey or GFSw and their mixtures, 0.25 to 10 mg/mL RE, and 0.0025 to 0.1 mg/mL CA or EP either individually or at the corresponding mixture concentrations.

### 2.5. Antioxidant Activity

#### 2.5.1. Radical Scavenging Activity by ABTS Assay

The antioxidant method of neutralizing the ABTS radical was determined by the ABTS radical cation discoloration test [21]. ABTS^+^ was produced by the reaction between 7 mM ABTS in water and 2.45 mM potassium persulfate, incubated in the dark at room temperature for 12–16 h before use. The ABTS^+^ solution was then diluted with 96% ethanol to have an absorbance of 0.700 ± 0.04 at 734 nm. A volume of 20 μL of sample or standard solution (prepared as described above, 2.4) was mixed with 180 μL of diluted ABTS^+^ solution, and the absorbance was measured at 734 nm after 30 min of incubation at room temperature.

#### 2.5.2. Radical Scavenging Activity by the DPPH Assay

DPPH (2,2-diphenyl-1-picrylhydrazyl) free radical-scavenging activity of the samples was performed according to [22] with some modification. Briefly, 100 μL of sample/standard solution was mixed with 100 μL of 0.3 mM DPPH solution in 99.6% (*v*/*v*) ethanol. The absorbance was read at λ = 517 nm after 30 min of reaction using a UV-Vis plate reader (CLARIOstar, BMG LABTECH, Ortenberg, Germany).

#### 2.5.3. Cupric-Ion Reducing Antioxidant Capacity (CUPRAC) Assay

The antioxidant method of cupric ion reducing capacity (CUPRAC) was performed according to a method adapted from [23] as follows. Ten microliters of sample/standard solutions were mixed with 30 µL CuSO_4_ (5 mM), 30 µL neocuproine (3.75 mM) and 280 µL distilled water, reaching a final volume of 350 µL. After 30 min, the absorbance was measured at λ = 450 nm. A calibration curve of Trolox as the standard substance was calculated based on several Trolox concentrations tested. The standard solutions started from a stock solution of 10 mM Trolox in 70% (*v*/*v*) ethanol and were used for the calibration curve within the concentration interval of 0–2 mM Trolox.

#### 2.5.4. Ferric-Ion Reducing Antioxidant Power (FRAP) Assay

The antioxidant method of ferric ion reducing power (FRAP) is based on the ability of antioxidants to reduce the tripyridyltriazine-Fe^3+^ (Fe (III)-TPTZ) complex to the blue-colored tripyridyltriazine-Fe^2+^ (Fe (II)-TPTZ) complex by the action of electron released by the antioxidant.

The determination of the antioxidant power of iron reduction was performed by the method described by [24] with some modifications. The FRAP reagent was prepared by mixing 10 parts of 0.3 M acetate buffer pH 3.6 with one part of 10 mM TPTZ (solubilized in 40 mM HCl) and one part of 20 mM FeCl_3_ solution (10:1:1). An aliquot of 15 µL of /standard solution was added to the 285 µL FRAP reagent. The absorbance was read at 593 nm after incubation for 30 min at 37 °C in the dark. A calibration curve of Trolox as the standard substance was calculated based on several Trolox concentrations tested. The calibration curve was made from the concentration range of 0–450 µM Trolox/mL in 70% (*v*/*v* ethanol).

#### 2.5.5. Evaluation of Modulation Activity between Honey/GFSw and Polyphenols

In order to establish possible modulations between honey/GFSw and polyphenols, the combination index and isobologram analyses were performed. For DPPH and ABTS, which presented non-linear effect dependence on concentration, the Webb analysis was also performed, in which the theoretical inhibited fraction was calculated by the formula: 100 − ((100 − *f_n,A_*) × (100 − *f_n,B_*)), where *f_n,A_* and *f_n,B_* represent non-inhibited fractions by *A* and *B* when tested individually, respectively. The combination index (CI) was calculated from the formula:

$$CI=\frac{C_{A,m}}{C_{A,i}}+\frac{C_{B,m}}{C_{B,i}}$$

*C_A,m_* and *C_B,m_* are the concentrations of *A* and respectively *B* in the mixture that give the same effect as the individual concentrations, *C_A,i_* and respectively *C_B,i_*. *A* and *B* represent the two components that are mixed. *A* was honey/GFSw and *B* was RE/CA/EP. Isobologramic diagrams were produced based on these values. The theory behind the methods is described in reference [25].

The CI and isobologram analysis were determined at IC_50_ and IC_20_ (50% and 20% substrate inhibition, respectively) in the case of DPPH and ABTS. These values were calculated based on the median-effect equation proposed by Chou group that transforms a non-linear dose-effect curve into a linear form:

$$\log(\frac{f_{i}}{f_{n}})=a\times \log(conc)+b$$

where *f_i_* and *f_n_* are the inhibited and non-inhibited fractions, respectively, *a* is the slope and *b* = −*a* × IC_50_. The non-inhibitory (*f_n_*) and inhibitory (*f_i_*) fractions were expressed as percent and calculated from the formula:

$$f_{n}=\frac{\left(A_{0}-blank_{0})-(A_{c}-blank_{c}\right)}{\left(A_{0}-blank_{0}\right)}\times 100$$

and *f_i_* = _100_ − *f_n_*, respectively, where *A*_0_ and blank 0 are the absorbances of the substrate in the absence of the antioxidant and of the corresponding blank (solvent without substrate), respectively, and *A_C_* and blank *C* are the absorbances of the substrate in the presence of concentration *C* of the antioxidant and of the corresponding blank (antioxidant without substrate), respectively. In the case of CUPRAC and FRAP, the Trolox calibration curve was used to express CI at 1 mM Trolox equivalent.

### 2.6. Preparation of Honey-Mimetic Natural Deep Eutectic Solvent

The natural deep eutectic solvent (NaDES), which mimics honey, was prepared based on the content of the main sugars in multifloral honey according to the literature data [26]. The NaDES was synthesized by mixing glucose, fructose, saccharose, and water (1:1.3:0.2:5 by molar ratios). The mixture was heated and stirred at 70 °C until a clear, viscous mixture was formed (≈2^1/2^ h). From this point forward, the NaDES formed, abbreviated as GFSw, was cooled to room temperature and kept in a closed bottle until use.

### 2.7. Physico-Chemical Characterisation of Honey-Mimetic Natural Deep Eutectic Solvent (GFSw) and Honey

#### 2.7.1. FTIR Analysis 

FTIR-ATR spectroscopy measurements were performed using a Spectrum GX spectrometer (Perkin Elmer, Beaconsfield, UK), applying the Attenuated Total Reflectance (ATR) technique with a diamond crystal, according to the manufacturer’s instructions. IR absorption spectra were obtained by the acquisition of 32 scans, with a resolution of 4 cm^−1^ in the region between 4000 and 600 cm^−1^. The spectra of GFSw were compared with honey.

#### 2.7.2. Thermogravimetric Analysis

Thermogravimetric analysis (TGA) was performed using a TA-Q5000 V3.13 (TA Instruments, Inc., New Castle, DE, USA) device with nitrogen as the purge gas at a 50 mL/min flow rate, according to the manufacturer’s instructions. The runs were carried out using a 10–15 mg sample in a platinum pan and a synthetic air atmosphere with 50 mL/min airflow. The temperature range was between 25–700 °C with a heating rate of 10 °C/min.

#### 2.7.3. Differential Scanning Calorimetry Analysis

Differential scanning calorimetry (DSC) analysis was performed using a DSC Q2000 (TA Instruments, Inc., New Castle, DE, USA) under helium flow (25 mL/min), according to the Manufacturer instructions. Samples weighing around 10 mg were packed in aluminum pans, and MDSC analysis was carried out to determine the thermodynamic parameters (transition temperature—Tg, specific heat capacity—ΔCp, enthalpy—ΔH) and the glass transition.

#### 2.7.4. Surface Tension Analysis

The surface tension of DES and honey was measured by optical tensiometer OCA 50EC (DataPhysics Instruments GmbH, Filderstadt, Germany), according to the manufacturer’s instructions. The method was based on evaluating the shape of a liquid droplet suspended at the needle end of a syringe. The diameter of the needle had an outer diameter of Φ = 1.83 mm, an inner diameter of Φ = 1.36 mm, and the length of the needle was l = 38.1 mm. The shape of the drop represents the result of the interfacial tension of the analyzed liquid (a spherical shape produces a minimum surface area) and the gravity (elongation of the drop due to the mass of the liquid). The Laplace-Young equation was used to determine the surface tension by software calculation.

#### 2.7.5. Measurement of Specific Density

The densities of the DES and honey samples were measured using a density meter Easy D40 (Mettler Toledo, Columbus, OH, USA), according to the manufacturer’s instructions. For each sample, three replicates were obtained, and the average was reported.

#### 2.7.6. Measurement of Water Activity 

Water activity was measured at 22 °C using LabMaster-aw neo (Novasina AG, Lachen, Switzerland) equipment, according to the manufacturer’s instructions. For each determination, four replicates were obtained, and the average was reported.

#### 2.7.7. Measurement of pH

The pH values of samples were measured using a pH-meter SevenCompact 2S10 (Mettler Toledo, Columbus, OH, USA), according to the manufacturer’s instructions.

#### 2.7.8. Measurement of Refractive Index and Total Soluble Solids

Refractive index and total soluble solids (TSS) were determined using a digital refractometer (MyBrix, Mettler Toledo, Columbus, OH, USA), according to the manufacturer’s instructions. The refractometer was first calibrated with double-distilled water. The total soluble solids of honey and GFSw were represented by total soluble sugar and expressed as Brix degrees (one percent of TSS is considered one ^0^Brix) [27]. For each determination, four replicates were obtained, and the average values were reported.

#### 2.7.9. Spray-Drying

Honey and GFSw were powdered by a spray-drying method. The honey solution was prepared for spray-drying according to [28] with some modification by mixing with maltodextrin (MD) and ddH_2_O to obtain a solution with 75% solids (*w*/*v*). The ratio between honey and MD was 60:40 (*w*:*w*). The GFSw solution was prepared in the same way. The spray drying of honey and of the GFSw solutions was performed using a Mini Spray Drier B—290 (Büchi, Flawil, Switzerland). The spray drier was also equipped with a pre-drying air module that worked in parallel during the drying process with the spray drier. During the spray drying process, the pre-drying air module showed a 69–72% dehumidification at 0–1 °C. Honey and GFSw solution was spray dried under the following conditions. The feed solution was introduced, along with the dehumidified drying air through a three-fluid nozzle system mounted on top of the spray drier, the inlet air drying temperature was set at 120 °C, and the debit of the peristaltic pump was set at 10% (3 mL/min). The debit flow meter of drying air was set at 55 mm (670 L/h, with a 1.05 bar pressure drop, meaning that the actual inserted air volume was 1374 L/h at standard temperature and pressure, as recorded in the instructions manual). During the spray-drying process, the outlet temperature was recorded at 50 °C for honey and 74 °C for GFSw. Powders were kept in a desiccator to prevent moisture.

#### 2.7.10. Scanning Electron Microscopy

Scanning electron microscopy (SEM) was performed with TM4000Plus II tabletop electron microscope (Hitachi, Tokyo, Japan) at 5 kV electron acceleration voltage, 200× and 600× magnification, backscattered-electron (BSE) detector, and standard (M) vacuum mode, according to the manufacturer’s instructions.

#### 2.7.11. X-ray Diffraction

X-ray diffractograms were obtained with a SmartLab diffractometer (Rigaku, Tokyo, Japan) in “parallel beam” geometry, using Cu-K_α_ radiation (𝜆 = 1.5406 Å) obtained at an acceleration voltage of 45 kV and emission current of 200 mA, and a scintillator detector, according to the manufacturer’s instructions. The diffractograms were recorded in the 2θ range of 5–90° in steps of 0.02° at a speed of 4°/min.

### 2.8. Preparation of GFSw with Raspberry Extract and with Polyphenolic Standards

The mixtures of GFSw and polyphenols were prepared in a similar way to the mixtures using honey described above. The extracts of raspberry were split equally into two fractions and were concentrated to dryness using a semi-automated evaporation system MultiVap54 (Lab tech, Sorisole, Italy) at 40 °C. One of the samples was resuspended in GFSw at a ratio of 1/20 (*w*/*w*), and the other one was resuspended in 70% ethanol solution at the same ratio of 1/20 (*w*/*v*). The extract was solubilized in GFSw using an ultrasonic bath, mixed thoroughly, and the polyphenols were left to diffuse overnight. The AOA of the samples was assayed using the same spectrophotometric methods: radical scavenging activity (ABTS and DPPH) and reducing antioxidant power (CUPRAC and FRAP).

The individual major polyphenols found to be in raspberry extract (caffeic acid and epicatechin) were solubilized in GFSw at the same concentration and in the same way for honey described above.

### 2.9. Statistical Analysis

We calculated confidence intervals at 95% confidence for the isobolographic analysis of the AOA activities. The confidence intervals were calculated by subtracting and adding the value 1.96 × SD/sqrt (*n*), where SD is the standard deviation and *n* is the number of measurement replicates (*n* = 3 in all cases).

## 3. Results

The polyphenolic composition of the raspberry extract was determined based on several assays: total polyphenolic content (TPC), total flavonoid content (TFC), total hydroxycinnamic content (HAT), total anthocyanin content (TAC), and HPLC analysis.

### 3.1. Screening of Bioactive Compounds in Honey and Raspberry Extract

#### 3.1.1. Total Polyphenols, Flavonoids, and Anthocyanins Content

The results of TPC, TFC, HAT, and TAC of raspberry and honey samples are summarized in Table 1.

The results indicated that the TPC, TFC, and HAT (282 ± 10.72 GAE mg/100 g DW, 29.88 ± 1.05 QE µg/g DW, and 57.92 ± 2.92 Chae mg/100 g DW) were significantly higher than that of honey (4.63 ± 0.30 GAE mg/100 g DW, 2.25 ± 0.057 QE µg/g DW and 2.89 ± 0.086 Chae mg/100 g DW). According to literature data [29,30,31], the concentration of phenolic compounds (which also includes polyphenols, flavonoids, hydrocinnamic acids, and anthocyanins) is dependent on numerous factors (species, cultivars, environmental, storage, methods of extractions, and analysis). For these reasons, the concentration of phenolic compounds varies in different scientific articles. Our results correspond to the literature. The value of TPC from raspberry was in the range of reported results by [32], who obtained a total phenolic content in the range 164.54–416.24 mg GAE/100 g, and also was higher than the results reported by [13] −140.31–160 mg/100 g FW. The total flavonoid content was lower than those revealed by [13], who obtained values in the range 88.98–111.14 mg/100 g. The total anthocyanin value was slightly higher than the values reported by [32,33] and lower than the values obtained by [13].

Our results for TPC and TFC were in agreement with literature data [34,35] concerning polyphenols in Romanian honey samples. Our value of TPC was lower than the values reported by [35] and slightly higher than the results reported by other authors [34]. These differences between our data and the data from the literature could be related to the composition of honey, which is affected by various factors, such as the floral and geographical origin, the collection season, the storage, and the harvesting technology.

#### 3.1.2. HPLC Analysis

The identification and quantification of phenolic acids and flavonoids in honey and raspberry were performed by an HPLC technique. In the case of the honey sample, the HPLC analysis of phenolic acids from the honey sample was performed after a preliminary isolation step of the phenolic compound by SPE from the honey matrix. The chromatograms of polyphenolic compounds from raspberry and honey samples are presented in Supplementary Materials (Figures S1–S4). The deconvolution of the peaks was performed in OriginPro 2018 (OriginLab Corporation, USA). The amounts of phenolic acids and flavonoids identified in honey and raspberry are summarized in Table 2.

Phenolic compounds found in the honey analyzed included 4-hydroxybenzoic acid, caffeic acid, p-coumaric acid, protocatechuic acid, ferulic acid, rutin, quercetin, apigenin, and myricetin. Overall, the concentration of phenolic compounds was in agreement with data reported in other scientific articles [34,35,36]. The content of protocatechuic acid, caffeic acid, myricetin, and 4-hydroxybenzoic acid in our honey sample was higher than the value reported by [34], who obtained 0.15, 0.14, and 0.50 mg/100 g, and 0.08 mg/100 g, respectively. They also reported the concentration of quercetin at 1.23 mg/100 g, which was lower than our value. The hydroalcoholic raspberry extract was analyzed to identify and quantify phenolic compounds. The phenolic compounds found in the raspberry extract were caffeic acid, ferulic acid, p-coumaric acid, epicatechin, rutin, quercetin, kaempferol, apigenin, and myricetin, and corresponded with literature data [13]. The highest content of phenolic acids analyzed was identified as caffeic acid with 770.96 ± 24.06 µg/g, and epicatechin with the highest flavonoid content at 1684.06 ± 77.88 µg/g.

### 3.2. Evaluation of the Antioxidant Activity of Honey and Its Formulations with Polyphenols

**Figure 1:**
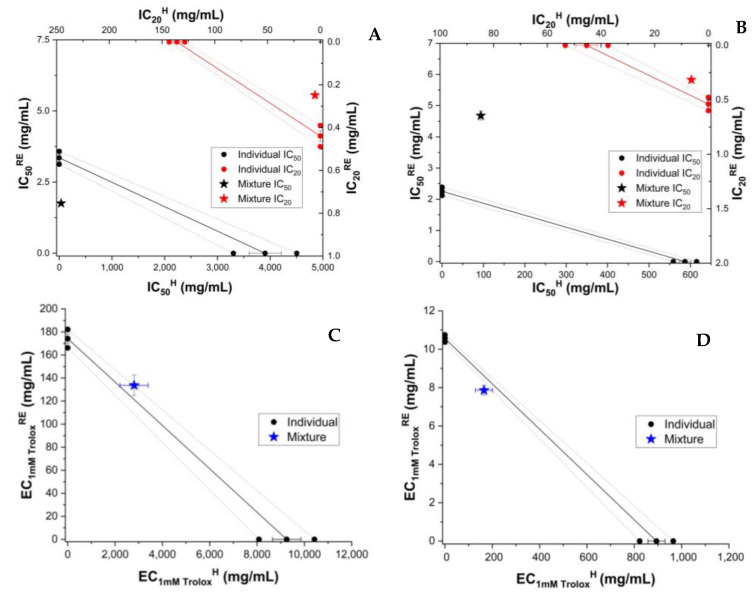
Isobolograms of honey (H) and raspberry extract (RE) based on IC_50_ (half-maximal inhibitory concentration) and IC_20_ (inhibitory concentration at 20% substrate inhibition) for DPPH (**A**), and ABTS (**B**) methods, and based on EC_1mM Trolox_ (effective concentration at 1 mM Trolox equivalent of the samples) for CUPRAC (**C**) and FRAP (**D**) methods. The error bars from three measurements are shown for each value. Confidence intervals at 95% confidence are shown by dashed lines.

According to our results, the AOA of honey enriched with raspberry extract was higher than commercial multifloral honey as determined by all methods (DPPH, ABTS, FRAP, and CUPRAC), as can been seen in the Supplementary Material Table S1. The concentration dependence of experimental and theoretical AOA of honey and RE (H_RE versus H + RE) is shown in Supplementary Information Table S2. As can be seen, the AOA varied linearly with concentration in the case of FRAP and CUPRAC and sigmoidal in the case of DPPH and ABTS. A similar trend occurred in the case of individual RE. Based on the individual calibration curves, isobologramic diagrams were built, as seen in Figure 1.

**Figure 2:**
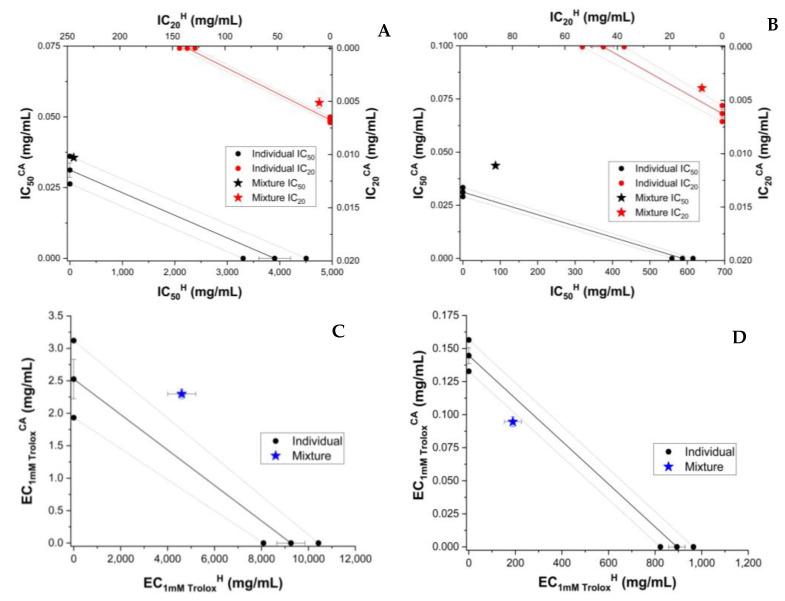
Isobolograms of honey (H) and caffeic acid (CA) based on IC_50_ (half-maximal inhibitory concentration) and IC_20_ (inhibitory concentration at 20% substrate inhibition) for DPPH (**A**) and ABTS (**B**) methods, and based on EC_1mM Trolox_ (effective concentration at 1 mM Trolox equivalent of the samples) for CUPRAC (**C**) and FRAP (**D**) methods. The error bars from three measurements are shown for each value. Confidence intervals at 95% confidence are shown by dashed lines.

**Figure 3:**
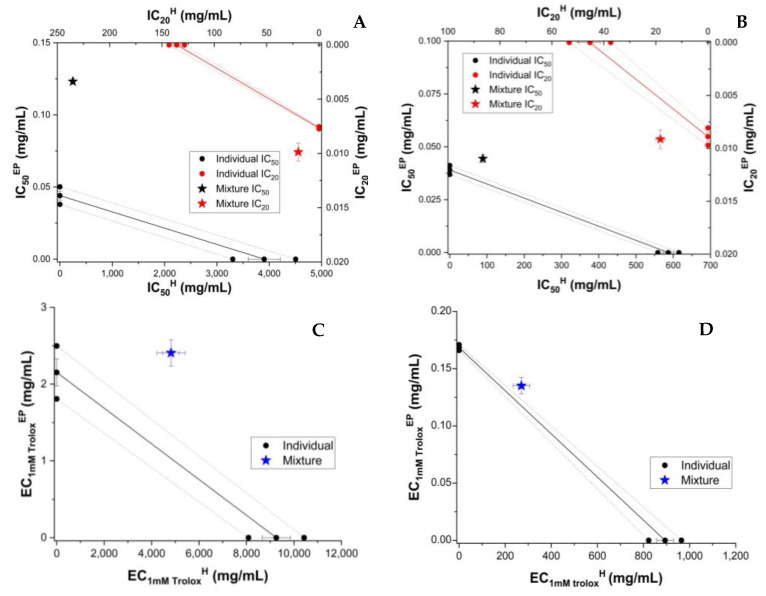
Isobolograms of honey (H) and epicatechin (EP) based on IC_50_ (half-maximal inhibitory concentration) and IC_20_ (inhibitory concentration at 20% substrate inhibition) for DPPH (**A**) and ABTS (**B**) methods, and based on EC_1mM Trolox_ (effective concentration at 1 mM Trolox equivalent of the samples) for CUPRAC (**C**) and FRAP (**D**) methods. The error bars from three measurements are shown for each value. Confidence intervals at 95% confidence are shown by dashed lines.

To compare the behaviour of RE with that of individual polyphenols dissolved in honey, we analyzed the AOA of the mixture of honey and individual major polyphenols from the raspberry extract. As shown above, HPLC analysis showed caffeic acid (CA) and epicatechin to be present in significant amounts. These were chosen to test the antioxidant behaviour induced by polyphenols and honey. For the determination of AOA activity, the polyphenol was resuspended in honey or 70% ethanol at the same polyphenol concentration (0.5 mg/g of honey, 0.5 mg/mL of 70% ethanol, respectively. Caffeic acid and epicatechin enhanced the AOA of honey. (Table S1). The concentration dependence of experimental and theoretical AOA of honey and the polyphenol (CA or EP) is shown in Supplementary Information Table S3. The concentration dependence of AOA was linear in the case of FRAP and CUPRAC and sigmoidal in the case of DPPH and ABTS (Supplementary Information Tables S1 and S3), as seen also for honey and honey with extract mixture (H_RE and H + RE). A similar trend was seen in the case of individual CA and EP. The results of isobolomic representations of honey enriched with CA and epicatechin (EP) are shown in Figure 2 and Figure 3, respectively.

The CI values were calculated for all combinations (Table 3). The CI values were dependent on method, dose and polyphenol type, and varied between a minimum of 0.532 (DPPH IC50 of H_RE) and 2.885 (DPPH IC50 of H_EP).

### 3.3. Comparison between Honey and the Honey-Mimetic NaDES, GFSw

We prepared a honey-mimetic NaDES based on the content of glucose, fructose, sucrose, and water according to the literature data [26]. This NaDES was characterized by different methods and was compared with wildflower honey.

**Figure 4:**
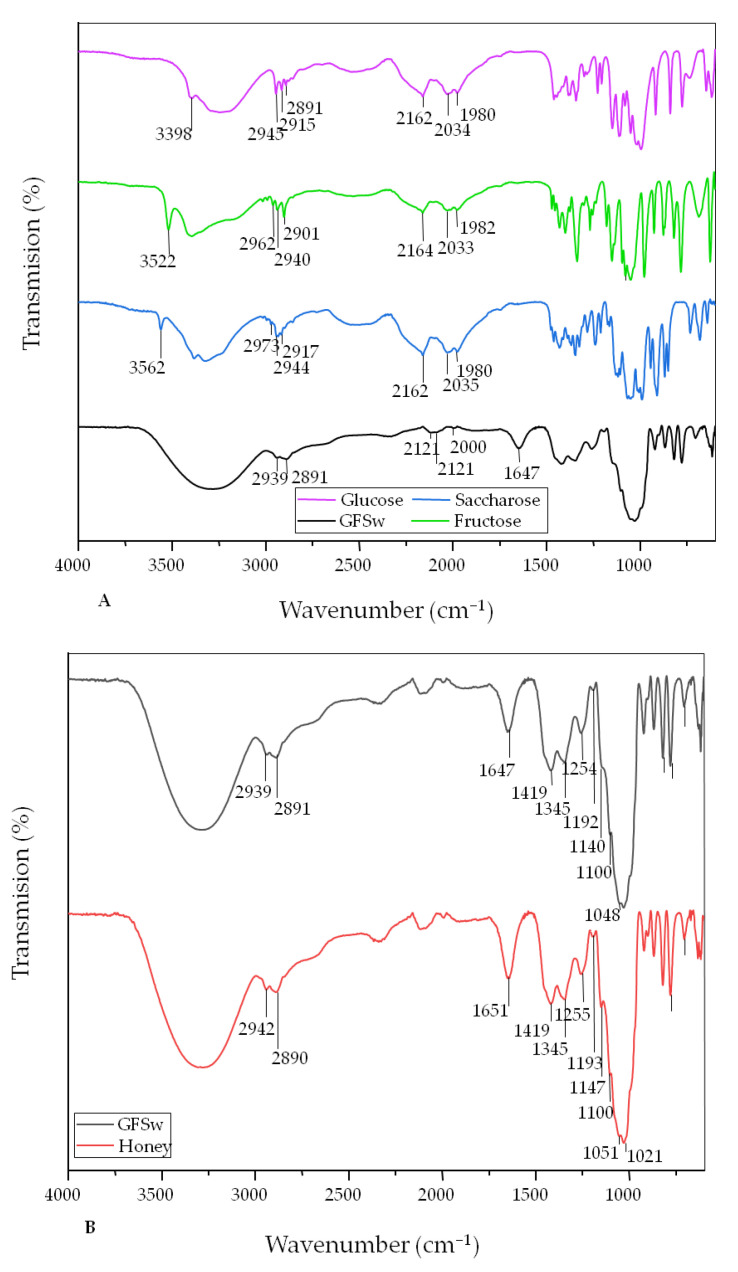
Comparison between the ATR-FTIR spectra of honey and the main components of GFSw—glucose, fructose, and sucrose (**A**) and between the ATR-FTIR spectra of honey and GFSw NaDES (**B**). The absorption bands from 1419 cm^−1^ and 1345 cm^−1^ are characteristic of the bending vibrations of O-CH and C- CH in the structure of carbohydrates or of the bending vibrations coming from OH from the C-OH group. The bands from about 1255–1140 cm^−1^ are characteristic of the stretching vibration of C-H or C-O from carbohydrates. The vibration with a maximum of about 1100 cm^−1^ is a band that can come from the C-O vibration in the C-O-C group. The bands of approximately 1055, 1025, 990, and 777 cm^−1^ can be assigned to C-O stretching from the C-OH group or C-C from the carbohydrate structure. The band at 987–988 cm^−1^ is characteristic of the glycosidic bond C-O-C. The spectral area from 898 to 818 cm^−1^ is characteristic of the anomeric vibrational region of carbohydrates or the C-H deformation group [37,38].

Fourier transform infrared spectroscopy (FTIR) was used to study the interaction between the main components of NaDES, to follow the structural changes induced by the formation of DES, and to compare NaDES with honey (Figure 4).

The spectra of the analyzed samples (4000 to 600 cm^−1^) show the characteristic band of hydrogen bonds at 3280 cm^−1^ (O-H hydrogen bonds), the bands around 2900 cm^−1^, characteristic of the stretching vibrations of the C-H groups, and the fingerprint region (1500–700 cm^−1^).

The FTIR spectra showed that there were significant changes upon mixing the three carbohydrates and water compared to individual compounds (Figure 4A). The most significant changes were in the fingerprint region, where the number of bands decreased in GFSw.

The individual carbohydrates were characterized by multiple sharp bands, while in GFSw, these sharp bands disappeared, and the remaining bands became broader. Some bands, such as the sharp band in the region 3400–3600 cm^−1^ and bands in the region 2300–2000 cm^−1^ that were present in the individual carbohydrates disappeared, and a new band appeared at 1647 cm^−1^ in GFSw. This band was present in honey as well (Figure 4B). The FTIR spectrum of GFSw was very similar to the spectrum of honey.

**Figure 5:**
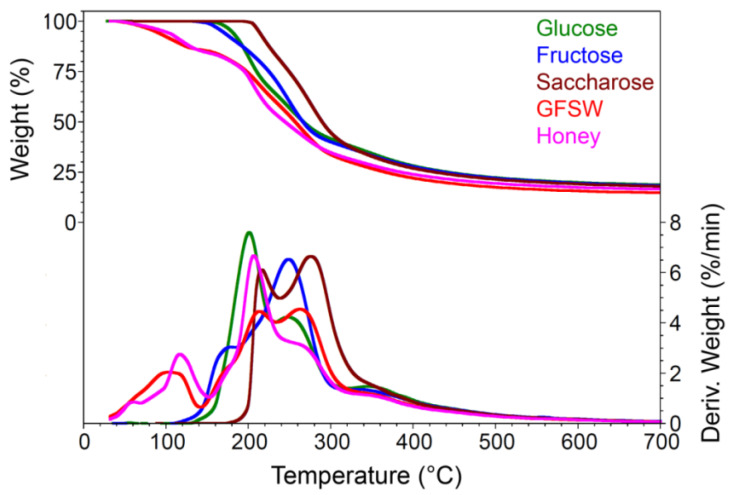
TGA/DTG curves of honey and DES mimetic with honey (GFSw,) and of the main components of GFSw (glucose, saccharose, fructose).

The TG/DTG curves of the samples (Figure 5) showed that the decomposition of honey occurred in consecutive events involving different stages.

These stages start at room temperature and end close to 600 ° C. The results are summarized in Table 4

The first and second transition of thermal decomposition took place between room temperature and about 140–155 °C, and can be attributed to the water loss and volatile constituents and possibly small contributions from protein denaturation; the last two only in the case of honey.

The next thermal events, which occurred between 155 and 700 °C, can be attributed to the thermal decomposition of sugars and materials resulting from caramelization processes.

The percentage of residues at 700 °C corresponded to the content of carbonaceous materials formed as a result of advanced pyrolysis of sugars.

The ash content values resulted from the combustion of carbonaceous materials and represent traces of inorganic materials or graphitized organic materials. Honey and GFSw showed decompositions within similar temperature ranges, but the weight losses presented some differences between the two products at transitions 2 and 3.

**Figure 6:**
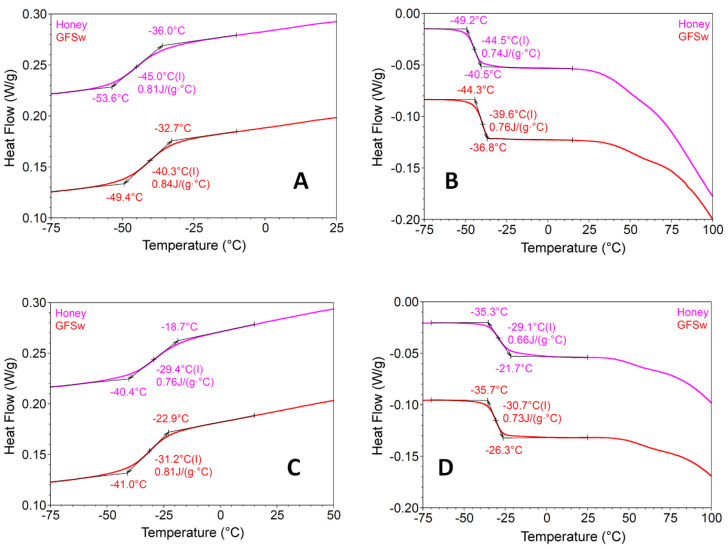
Differential scanning calorimetry (DSC) heating thermograms of honey and GFSw. First cooling cycle in the range of temperatures from 0 °C to −75 °C (**A**), first heating cycle in the range of temperatures from −75°C to 100 °C (**B**), second cooling cycle in the range of temperatures from 0 °C to −75 °C (**C**), second heating cycle in the range of temperatures from −80 °C to 100 °C (**D**).

Figure 6 shows DSC thermograms obtained after cooling and heating cycles of honey and GFSw samples. The DSC analysis of the first cooling from room temperature to −75 °C showed a glass transition for honey (from −36.0 °C to −53.6 °C, with a mid-point, glass transition temperature (Tg) of −45.0 °C) and GFSw (from −32.7 to −49.4 °C, with Tg of −40.3 °C). The specific heat capacity was similar between honey (0.81 J/(g °C) and GFSw (0.84 J/(g °C).

The corresponding enthalpy was 235.5 J/g for honey and 243.5 J/g for GFSw. This transition occurs when the material changes upon cooling from the rubber-like state into the hard, glassy state [39]. The first heating from −75 °C to 100 °C presented similar reversible transitions, but with less negative temperatures and lower heat capacities, representing transitions from the glassy solid state to the rubbery state upon heating. There was also a change in heat flow at high temperatures (>25 °C), steeper and larger for honey than for GFSw.

In the case of the second cooling and second heating, the mid-point temperatures of both honey and GFSw become less negative compared with the first transitions and much closer between the two samples, but with changing order (mid-point temperatures of GFSw are lower than mid-point temperatures of honey). The temperature differences (ΔT) of Tg values between the first and second transitions were higher with approx. 6.5 °C for honey than for GFSw. The change in heat flow at high temperatures also became similar between the two samples and less pronounced than during the first transitions.

**Figure 7:**
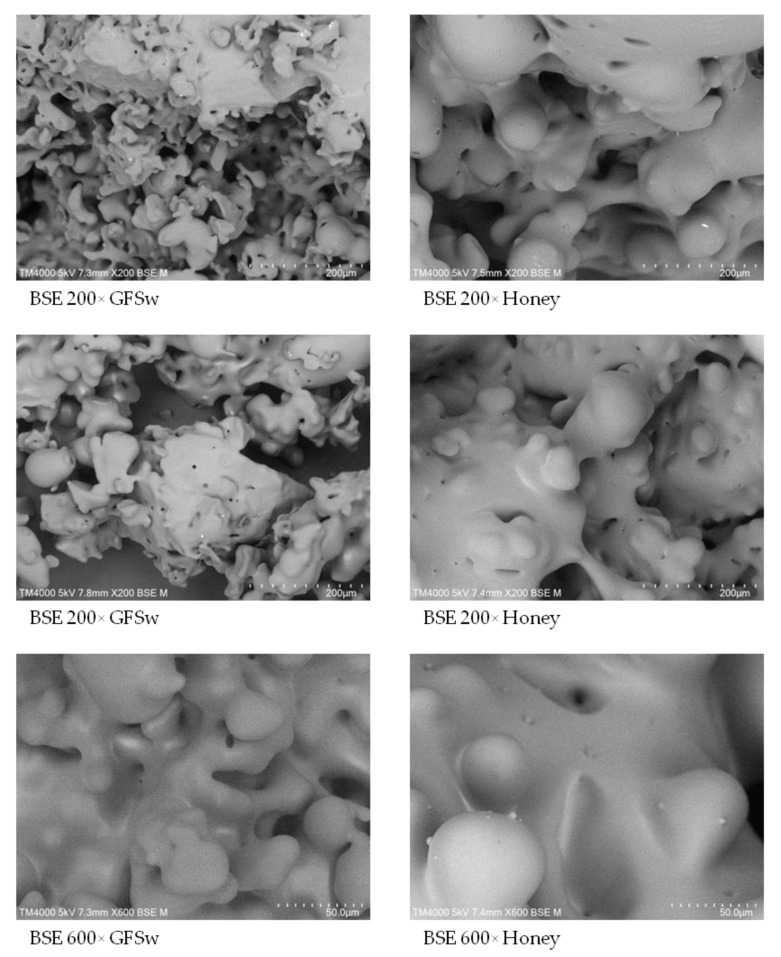
SEM micrographs of spray-dried honey and GFSw at 200× and 600× magnification.

We next wanted to obtain information about the morphological and structural behavior of GFSw compared to honey. Honey and, respectively, GFSw, were spray-dried after mixing with maltodextrin and investigated by SEM and X-ray diffraction techniques. Figure 7 shows several SEM micrographs of the external microstructure of honey and GFSw powders at 200× and 600× magnification. As can been seen, the products show similar morphological features, with smooth surfaces and aggregates of round microparticles with linkages between them, similar to other types of honey reported [28]. There are some small holes present in both honey and GFSw clearly visible at 600× magnification. Although similar, the particles of honey seem to be larger than the particles of GFSw, and we observed additional very small particles on the honey surface, which could be due to the presence of other compounds in honey.

**Figure 8:**
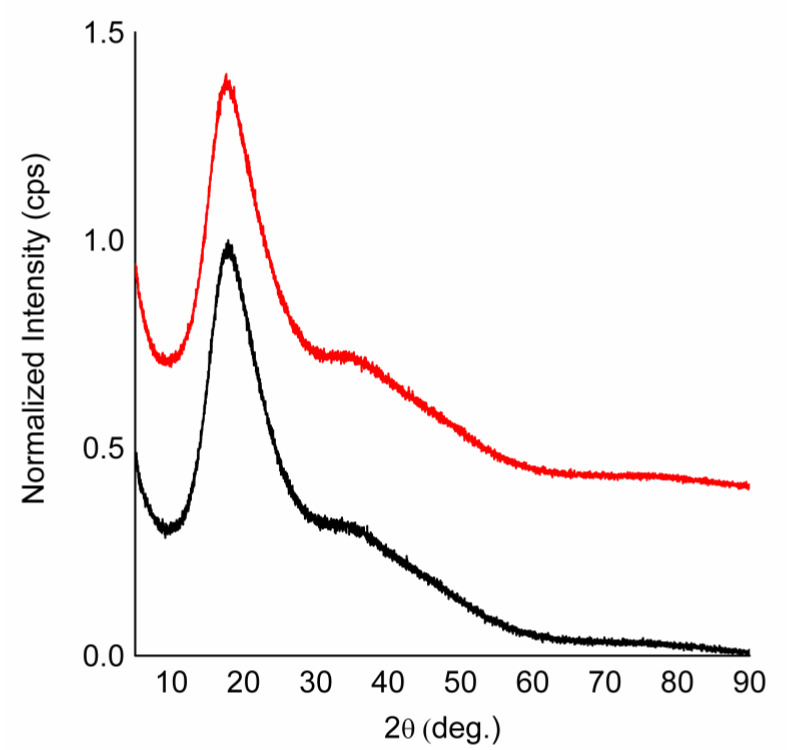
X-ray diffractograms of honey (red) and GFSw (black).

The XRD profiles show two wide main peaks and a much less intense one, similar to honey and GFSw (Figure 8).

The diffractograms could be deconvoluted by Gaussian decomposition in up to four apparent peaks, at approx. 2θ = 17.58°/17.8°, 35.9°/35.01°, 44.2°/46.2°, and 78.3°/78.1°, for honey/GFSw but with the two intermediate peaks overlapping significantly and the fourth (78°) with a very small amplitude (Supplementary Figures S5 and S6, Table 3).

When fitting with two maxima, the 2θ were approx. 17.59°/17.72° and 36.16°/34.53° for honey/GFSw. The fitting were slightly but not significantly improved from 2 to 4 angles. The corresponding average atomic distances resulting from 2θ were similar between honey and GFSw (Table 5).

### 3.4. Physicochemical Characteristics of Honey and GFSw

The physicochemical characteristics of surface tension, density, pH, water activity, refractive index, and Brix degree of honey (total soluble solids—TSS) and GFSw are summarized in Table 6. Most physicochemical parameters measured (surface tension, density, pH, refractive index, and TSS) had slightly lower values in the case of honey than in the case of GFSw. Water activity was slightly higher in the case of honey compared with GFSw.

**Figure 9:**
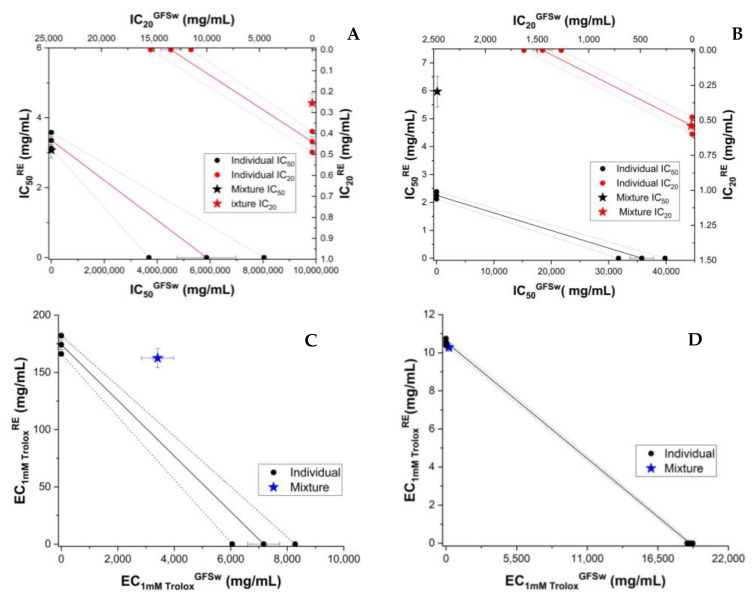
Isobolograms of NaDES (GFSw) and raspberry extract (RE) based on IC_50_ (half-maximal inhibitory concentration) and IC_20_ (inhibitory concentration at 20% substrate inhibition) for DPPH (**A**), and ABTS (**B**) methods, and based on EC_1mM Trolox_ (effective concentration at 1 mM Trolox equivalent of the samples) for CUPRAC (**C**) and FRAP (**D**) methods. The error bars from three measurements are shown for each value. Confidence intervals at 95% confidence are shown by dashed lines.

**Figure 10:**
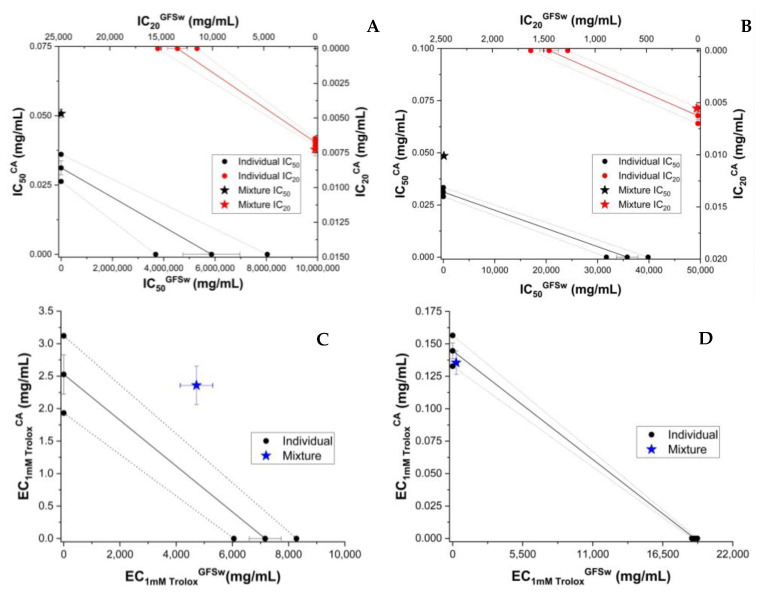
Isobolograms of NaDES (GFSw) and caffeic acid (CA) based on IC_50_ (half-maximal inhibitory concentration) and IC_20_ (inhibitory concentration at 20% substrate inhibition) for DPPH (**A**), and ABTS (**B**) methods, and based on EC_1mM Trolox_ (effective concentration at 1 mM Trolox equivalent of the samples) for CUPRAC (**C**) and FRAP (**D**) methods. The error bars from three measurements are shown for each value. Confidence intervals at 95% confidence are shown by dashed lines.

**Figure 11:**
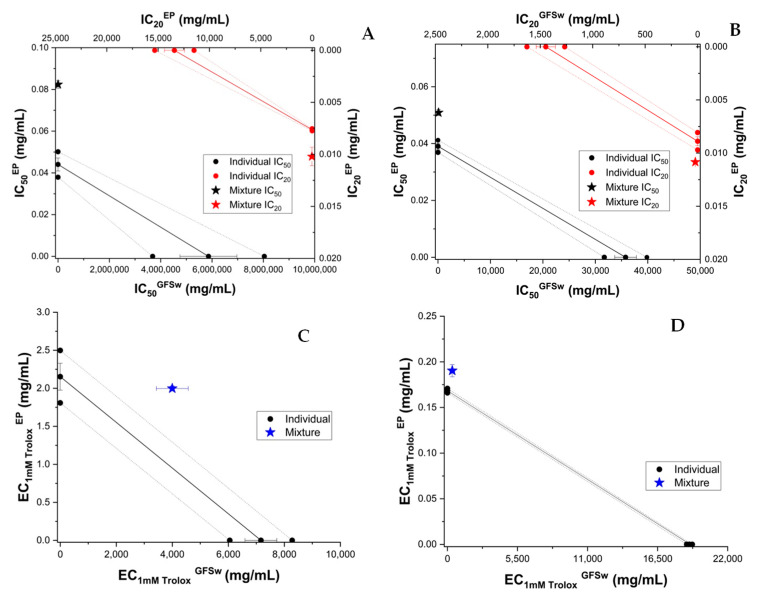
Isobolograms of NaDES (GFSw) and epicatechin (EP) based on IC_50_ (half-maximal inhibitory concentration) and IC_20_ (inhibitory concentration at 20% substrate inhibition) for DPPH (**A**), and ABTS (**B**) methods, and based on EC_1mM Trolox_ (effective concentration at 1 mM Trolox equivalent of the samples) for CUPRAC (**C**) and FRAP (**D**) methods. The error bars from three measurements are shown for each value. Confidence intervals at 95% confidence are shown by dashed lines.

We wanted to check if the mixtures GFSw—extract and GFSw—polyphenol standard had the same behaviour as in the case of honey. The NaDES GFSw was enriched with concentrated raspberry extract/standard (caffeic acid or epicatechin) in the same ratio of 1:20 (*w*/*w*) as with honey. The concentration dependence of AOA was similar in the case of GFSw compared to honey (linear for FRAP and CUPRAC and sigmoidal for DPPH and ABTS: Tables S4 and S5), but GFSw had very small AOA activity, as expected due to the lack of polyphenols, except in the case of CUPRAC method, where GFSw activity was similar to the honey activity. The isobologram diagrams are presented in Figure 9, Figure 10 and Figure 11 for RE, CA and EP, respectively. The CI values are shown in Table 7.

The GFSw values that resulted in the same effect as the mixture were extremely high and non-realistic (especially with the DPPH method). This suggests that in this case, NaDES acts more as potentiator or inhibitor rather than synergiser or antagoniser, because of its lack of AOA activity.

The CI values were dependent on method, dose and polyphenols type, and varied between a minimum of 0.572 (DPPH IC_20_ of GFSw_RE) and 2.646 (ABTS IC_50_ of GFSw_EP).

## 4. Discussion

As mentioned previously, phenolic compounds are those most responsible for the antioxidant activity of honey and plant extracts. The antioxidant properties of phenolic compounds are attributed to their capacity to neutralize free radicals by several mechanisms, such as HAT (hydrogen atom transfer), SET-PT (single electron transfer via proton transfer), sequential proton loss electron transfer or TMC (transition metal chelation) [40].

The AOA of the samples (honey and raspberry extract) could be correlated with the concentrations and profiles of polyphenols analyzed by colourimetric assays (TPC, TFC, HAT) and the HPLC method. As expected, the AOA of honey enriched in raspberry polyphenolic extracts was much higher than the AOA of pure honey at all concentrations tested, irrespective of the AOA method employed.

It has often been claimed that honey constituents, as well as mixtures of honey with other ingredients, act or induce synergistic effects. Still, these claims have not been thoroughly and rigorously investigated and proven. In some studies, synergism was less evident, but we believe this was partially due to the method of evaluation approached [5,6]. In other cases, synergism seemed to depend on the honey type and the AOA method [8].

To the best of our knowledge, we performed here for the first time an investigation on honey—polyphenols and honey biomimetic NaDES—polyphenols modulations as reflected in AOA activities, based on isobolomic and combination index (CI) calculation, which represents a rigorous assessment of synergism or antagonism behaviour. Depending on the values of CI, the activity is theoretically defined as synergic (CI < 1), additive (CI = 1) and antagonistic (CI > 1). In practice, a confidence interval also applies and the values between 0.9 and 1.1 are usually considered as reflecting additive behaviour. The behaviour of RE was highly heterogeneous, varying from relatively strong synergism (CI = 0.532 for DPPH IC_50_) to relatively strong antagonism (CI = 2.237 for ABTS IC_50_), depending on the method and, in the case of DPPH and ABTS on the dose of mixture tested (Table 3). FRAP and CUPRAC showed additive behaviour, which correlated to the experimental and theoretical calibration curves (Table S2). The individual main polyphenols representative of phenolic acids and flavonoids (caffeic acid and epicatechin, respectively) from the extract had heterogeneous behaviour that depended on method and dose in a similar manner to that in the case of CA, and in a different manner to that in the case of EP, compared to RE. CA had a more heterogeneous behaviour than EP, the latter resulting in only different degrees of antagonism and no synergism. The ABTS activity of H_RE and H_CA seem to depend strongly on the dose applied, shifting from synergism at low doses to antagonism at high doses. The CI correlated with the experimental and theoretical calibration curves (Table S3).

A previous study from more than a decade ago applied a comparison approach, using ORAC and EPR techniques and physiologically relevant media [41]. It was found that certain combinations of antioxidant compounds at lower concentrations than in our study showed synergic effects, especially when involving sugar solutions and ascorbic acid together with polyphenols. Some results were contradictory, and further investigations are necessary but have not become available.

More recent work found other contradictory results with respect to sugar’s influence on polyphenol activity, with either synergetic or antagonistic behavior or no effect. This depended on compound and sugar types, concentration, and AOA method [42,43,44]. Based on some studies, a possible explanation for the synergetic effect observed could be related to the stabilization and protection of some polyphenols by sugars and vice versa [45,46,47]. Still, considering the heterogeneous behavior observed by several groups, this aspect probably has only a partial contribution and prevails only in specific cases, not as a general rule. Sucrose, glucose, and other sugars, for example, were previously shown to be able to quench ·OH radicals [48,49], and it was predicted that sucrose, sucrose radicals, and other sugars could interact with secondary metabolites, such as phenolic compounds, which could determine in some cases sucrose recycling [44]. It is possible that these features play a role in some particular situations, especially involving ·OH radicals. Sugar interaction with aromatic molecules was predicted based on molecular dynamics simulation and NMR, which showed that the interaction is rather hydrophobic in nature (sugar and aromatic rings stacking) than H-bonds driven [50,51].

Honey is considered an example of a natural deep eutectic solvent [2,52]. Most of the physicochemical and structural properties of our honey-mimetic NaDES were similar to those of honey. The FTIR spectral changes observed upon saccharide mixing compared to individual compounds indicate a shift from the crystalline nature characteristic of the saccharide powder to an amorphous structure within NaDES. These differences between crystalline and amorphous carbohydrates/dried melt samples have been reported before [53,54].

Similar ATR-FTIR spectra for honey, as obtained in our study, have been previously reported [28,55,56,57]. The results prove that the carbohydrate arrangement within GFSw is very similar to that in honey. The appearance of the band at 1640 cm^−^^1^ can result from the deformation vibrations of the −OH groups of the water present in GFSw. This band is very similar to the one from honey, indicating similar composition and molecular arrangement between GFSw and honey.

Based on XRD analysis, honey was previously characterized as having an amorphous structure. In accordance with the FTIR data, the XRD profile was characterized by broad diffraction maxima, indicating amorphous structures with short-range order (Figure 9). From the Gaussian decomposition of diffractograms, the main average distances between atoms in molecules were found to be similar between honey and GFSw. Few in-depth studies have reported on the diffractogram deconvolution and analysis of honey powders obtained by spray drying. A recent study reported similar results, with a four-peak Gaussian deconvolution profile, but with one difference, i.e., almost half 2θ angle (approx. 23°) compared with our result (approx. 45°). It is unclear at the moment what the cause of this difference is, but we believe that this angle has no physical significance as it did not significantly influence the overall results. As mentioned, there was a significant overlap of peaks 2 and 3, and the second angle (approx. 35°) had similar values between the two-peak fitting and four-peak fitting (Table 3). This aspect needs more in-depth studies and is beyond the purpose of this work. The most important outcome is that honey and GFSw gave very similar diffractograms, showing that both samples have a similar amorphous structure characterized by short-range order and only slightly different in the main distances between atoms [28].

Some differences were expected, such as pH slightly lower in honey than in GFSw due to the presence of organic acids and other molecules, or slightly lower surface tension of honey compared to GFSw due to more complex/slightly different composition. The pH range of honey is 3.5–5.5, and is influenced by various intrinsic and extrinsic factors [58]. The pH of GFSw is within this range. Surface tension is a measure of the interaction strength between the components in a sample. A better understanding of the intermolecular forces that are manifested in the liquid and between the surfaces is obtained from surface tension values [59]. The factors that influence the intermolecular forces within DES are temperature, the nature of HBA/HBD (hydrogen bond acceptor/hydrogen bond donor), and the molar ratio of the components (higher intake of HBA will increase the surface tension of the mixture) [60]. Honey contains about 80% solid components that melt individually above 100 °C (glucose, fructose, sucrose), and about 15–20% of water. The fact that this mixture is liquid at room temperature is due to the optimal combination ratio of the components. Honey has high surface tension due to the hydrogen bonds that are formed between saccharides and water (saccharides have many O atoms with non-participating e^−^ pairs that participate in the formation of H bonds with H from water), as well as cohesive forces. The higher surface tension in GFSw than in honey could indicate slightly stronger H-bond interactions in GFSw than in honey and/or higher percent of saccharides, which correlate with the higher density and lower water activity, respectively. Considering that TSS was similarly higher in GFSw than in honey, it is possible that a significant contribution comes from the slightly higher percent of saccharides in GFSw than in honey.

The more complex/slightly different composition of honey compared to GFSw is probably responsible also for the differences in the morphological features observed in SEM micrographs, honey resulting in larger spray-dried particles than GFSw.

Other minor differences between honey and GFSw correlated with each other, which shows that the data were consistent. For example, the experimental water content difference between honey and GFSw was similar to that determined by TSS (Δ = 2%) and TGA (Δ = 1.7%). The absolute values were lower (with approx. 4% water content) as determined by TGA compared to TSS for both honey and GFSw, which could represent molecules of water more tightly bound than the rest and which evaporated at higher temperatures. The water activity of honey was approx. 5.6% higher than that of GFSw, implying some water molecules are less tightly bound in honey than in GFSw, besides the contribution of the 2% higher water content in honey than in GFSw. Water activity is a quality parameter that is used to estimate the shelf life and crystallization rate of honey samples. In honey, water activity is influenced by sugar content (glucose, fructose, and other sugars) [61]. Refractive index, density, and TSS correlated with each other for both honey and GFSw.

The TGA profiles of honey and GFSw were similar, with small differences coming from the more complex composition of honey. Below 150 °C, the thermal profile of the tested honey was more complex than that of GFSw, with three apparent transitions compared to one, respectively. It was previously found that the profile in this region depends on the bee species and varieties of honey [62,63]. The differences in this region most probably come from the various volatiles and protein content in honey. The main transitions (between 150 and 340 °C) showed some difference between honey and GFSw, suggesting a higher amount of glucose in honey than in GFSw.

The thermodynamic behavior evidenced by DSC was similar between honey and GFSw, reflecting similar supramolecular structures, as suggested for other honey biomimetic NaDES previously obtained [2]. The water content and water activity correlated with the glass transition temperature (Tg) determined by DSC. The glass transition and specific heat capacity of honey were approx. 5 °C and 0.3 J/(g·°C), respectively, lower than those of GFSw both at first cooling and first heating. Lower Tg means honey freezes harder than GFSw, and this is correlated with the higher water activity, which assures higher plasticity and dynamics [64]. The steeper and larger transition above 25 °C at first heating and the higher ΔT (15.6 °C/15.4 °C versus 9.1 °C/8.9 °C) between the first and second cooling/heating for honey compared to GFSw is also related to water content and water activity. Honey loses water more easily and in higher amount than GFSw, which is reflected in a more significant change in Tg. After water loss, the Tg order was reversed. GFSw than had lower values than honey, which means that in the absence of water, GFSw is more dynamic than honey. The small differences in water content and water activity also correlate with the observation that the temperature difference between the inlet and outlet temperature during the spray-drying process was higher for honey (70 °C) than for GFSw (46 °C). These small variations in some properties and behavior most probably do not have a significant effect on the AOA behavior, so we believe that our GFSw NaDES is a close mimetic of honey, at least in this respect.

The honey-biomimetic NaDES obtained, GFSw, had a similar effect as honey on the AOA of raspberry extract and individual polyphenols (Figure 9, Figure 10 and Figure 11 versus Figure 1, Figure 2 and Figure 3 and Table 7 versus Table 3), but in the case of RE and CA honey showed, in general, a higher tendency towards synergism and less antagonism than GFSw. We call the phenomena observed as synergism and antagonism instead of potentiation/inhibition because both GFSw and especially honey present some AOA activity, although very low in the case of GFSw, except for the CUPRAC method. For convenience of comparison between honey and GFSw, we show the figures combined for each AOA method in Supplementary Material Figures S7–S10. We gathered all CI values within Table 8 and codified in Table 9 the CI value intervals as follows: 0.5–0.7 (+2, strong synergism); 0.7–0.9 (+1, moderate synergism); 0.9–1.1 (0, almost additive); 1.1–1.5 (−1, moderate antagonism); 1.5–2 (−2, moderate—strong antagonism); >2 (−3, strong antagonism). The color code indicates that most combinations presented similar behaviour of honey compared to GFSw.

As can been seen in Table 8 and Table 9, seven of thirty-six cases (less than 20%) were apparently additive. From the additive ones, half had a tendency towards synergism and half had a tendency towards antagonism. Approximately 20% of cases were clearly synergistic. The majority of cases (70%) from Table 8 are either antagonistic or additive tending to antagonistic. Most synergistic effects were seen in the DPPH and ABTS methods, but only at 20% inhibition (i.e., at lower concentrations of mixtures). At 50% inhibition (higher concentrations of mixtures) DPPH and ABTS present more antagonist cases than FRAP and CUPRAC. This dependence on mixture concentration in the case of DPPH and ABTS is probably related to inhibition caused by honey/GFSw, which could have several explanations, such as higher viscosity, too strong H-bonds and hydrophobic interactions between NaDES and polyphenols, change of redox potential or even increased competition of the weaker antioxidant (honey/GFSw) against the stronger antioxidant (polyphenols). From the cases that are antagonistic, some could probably become additive or even synergistic by modulating the honey: polyphenols ratio.

ABTS and DPPH RE differ in their behaviour with respect to the individual polyphenols. While in the case of ABTS the degree of effect (either synergistic, additive or antagonistic) seems to be cumulative in RE. In DPPH there seems to be an additional synergic interaction between polyphenols in mixture (extract) besides the effect induced by honey/GFSw. In other words, the polyphenols probably synergise each other in the DPPH, but not in the ABTS reaction.

Similar difference can be observed when comparing the two methods that gave linear dependence on concentration (CUPRAC and FRAP), with a relatively cumulative effect in FRAP and a polyphenol—polyphenol synergic effect that compensates for the antagonism between honey/GFSw and individual polyphenols in CUPRAC.

All in all, the data suggest that the polyphenols synergise each other in CUPRAC and DPPH methods, but not in FRAP and ABTS methods.

As mentioned above, for RE and CA, honey induces slightly lower CI than GFSw in general. This could be related, on one hand, to the presence of additional polyphenols that increase the synergism, and on the other hand to other elements present in honey that could synergise/potentiate the reactions more. The polyphenols and/or other elements present in honey seem to synergise CA in most of the cases, but not EP. Further investigations are needed in order to determine the elements in honey responsible for this difference. However, in general, our data show that honey mimetic mixtures of sugars behave similarly to honey, especially when antagonism is present, and in some cases no other compounds are necessary for a certain degree of synergic effect.

A recent study showed that some deep eutectic solvents based on ethylene glycol and choline chloride (ethaline) and, respectively, betaine and citric acid (BCA) can change the redox potential of polyphenols to lower values, and this is influenced by the composition of the solvents [65]. Lower redox potential implies that the polyphenols are more easily oxidized, so they have higher antioxidant capacity. Moreover, BCA was very efficient in stabilizing polyphenols. Honey-biomimetic NaDES was previously shown to improve the bioavailability, bioactivity and heat stability of compounds from *Astragali Radix*, a traditional Chinese medicine and functional food [2]. Taken together, these data imply that NaDES in general could modulate the AOA of polyphenols at different degrees, which could explain the behaviour observed in our study. The exact mechanism for each individual case remains to be established, requiring more in-depth analysis. The effect on redox potential could be one explanation for the heterogeneous behaviour observed, which was dependent on polyphenol type and AOA method.

Taken together, there are several possible mechanisms for the synergism/potentiation observed in some cases: stabilisation of polyphenols, redox potential, crowding space inducing environment by honey/GFSw, presence of enzymes in honey, multiple synergism between polyphenols and other compounds present in honey and extract, among others.

The behaviour seen in our mixtures is characteristic for what are called complex systems, in which unexpected behaviours manifest as a result of multiple interactions.

Some preliminary unpublished results from our group suggest that other extracts such as propolis or sea buckthorn extracts have similar behaviour when mixed with honey, which suggests that there are some general features that manifest independently of the extract type. Multiple functional foods or bioproducts for different biomedical fields based on honey enriched in extracted poplyphenols could be developed. The compositions would need to be optimized in order to reduce antagonism/inhibition and maximize the synergism. The applications will depend on multiple parameters, including the sensory one mentioned in the Introduction, but the synergic AOA will be beneficial in all cases. More work is needed to take into consideration optimizing the extract concentrations, extract—honey ratios, extract composition, honey type and properties and other parameters. Although our developed product does not present synergism in all cases, it still has advantages over simple honey, especially in the cases where hydroalcoholic supplements are forbidden.

## 5. Conclusions

We obtained honey enriched in polyphenols from raspberry extracts with conserved honey characteristics and enhanced (in some cases) synergic AOA between honey and polyphenols. The honey-biomimetic NaDES with similar properties as honey resulted in similar AOA behavior to honey when mixed with polyphenols, but honey seemed to have additional properties that increase synergism/reduce antagonism in some cases. The AOA behaviour of honey—polyphenols mixtures can be influenced by the AOA method, polyphenol type, mixture concentration and is characteristic for complex systems. The new product can be further optimized to maximize synergism, tested for biological activities and represents a promising functional food with enhanced AOA.

## Figures and Tables

**Table 1:** The results of total polyphenol content (TPC), total flavonoid content (TFC), total anthocyanin content (TAC).

	TPC GAE mg/100 g DW	TFC QE mg/100 g DW	HAT Chae mg/100 g DW	TAC mg cya, 3-Glu equivalent/100 g DW
Raspberry	282 ± 10.72	29.88 ± 1.05	57.92 ± 2.92	62.92 ± 0.64
Honey	4.63 ± 0.30	2.25 ± 0.057	2.89 ± 0.086	-

Values are mean ± SD (*n* = 3). GAE—gallic acid equivalent, QE—quercetin equivalent, Chae—chlorogenic acid equivalent, Cya3-Glu—cyaniding 3-glucoside, DW—dry weight.

**Table 2:** Polyphenols (phenolic acids and flavonoids) from raspberry and honey by HPLC analysis.

Polyphenols	Raspberry, µg/g	Honey, µg/g
**Phenolic acids:**		
Caffeic acid	770.96 ± 24.06	1.88 ± 0.02
Ferulic acid	7.14 ± 0.57	26.60 ± 1.46
p-coumaric acid	2.72 ± 0.39	3.06 ± 0.05
4-hydroxybenzoic acid	-	6.39 ± 0.09
Protocatechuic acid	-	2.66 ± 0.15
**Flavonoids:**		
Epicatechine	1684.06 ± 77.88	-
Rutin	83.43 ± 1.58	1.60 ± 0.03
Quercetin	16.12 ± 1.31	6.50 ± 0.44
Kaempferol	27.73 ± 2.09	-
Apigenin	22.44 ± 0.28	13.27 ± 0.39
Myricetin	-	8.39 ± 0.27

**Table 3:** Combination index (CI) between samples in AOA assays.

AOA Method	H_RE	H_CA	H_EP
FRAP	0.929 ± 0.029	0.866 ±0.021	1.104 ± 0.071
CUPRAC	1.069 ± 0.059	1.426 ± 0.016	1.641 ± 0.086
DPPH IC_50_	0.532 ± 0.003	1.174 ± 0.083	2.885 ± 0.183
DPPH IC_20_	0.604 ± 0.019	0.836 ± 0.030	1.436 ± 0.110
ABTS IC_50_	2.237 ± 0.043	1.552 ±0.081	1.292 ± 0.079
ABTS IC_20_	0.731 ± 0.032	0.790 ± 0.026	1.438 ± 0.055

H_RE-mixture of honey and raspberry extract, H_CA-mixture of honey-caffeic acid, H_EP—mixture of honey-epicatehin. IC_50_ and IC_20_ represent the analysis for doses at 50% and 20% substrate inhibition, respectively.

**Table 4:** TGA/DTG analysis of honey and GFS.

Sample	Transition	Temperature Range		Wt. Loss	Tmax
		(°C)		(%)	(°C)
Honey	1	32.7	153.7	15.96	117.5
	2	153.7	257.5	37.86	170.2/208.1
	3	257.5	338.0	16.26	273.6
	4	338.0	700.0	13.48	359.7
	Residue			16.44	(700 °C/N_2_)
	Ash			0.08	(700 °C/Air)
GFSw	1	32.7	142.6	14.26	104.8
	2	142.6	236.0	26.39	169.1/214.8
	3	236.0	341.7	31.43	264.3
	4	341.7	700.0	13.28	360.2
	Residue			14.63	(700 °C/N_2_)
	Ash			0.03	(700 °C/Air)

**Table 5:** XRD parameters from the Gaussian deconvolution of diffractograms for honey and GFSw.

**2 Peaks Deconvolution**		
**Sample**	**2θ**	**Distance (d)**
Honey	17.59°; 36.16°	5.04 Å; 2.48 Å
GFSw	17.72°; 34.53°	5.00 Å; 2.59 Å
**4 Peaks Deconvolution**		
Honey	17.58°; 35.9°; 44.2°; 78.3°	5.04 Å; 2.50 Å; 2.05 Å; 1.22 Å
GFSw	17.8°; 35.01°; 46.2°; 78.1°	4.99 Å; 2.56 Å; 1.97 Å; 1.22 Å

**Table 6:** Surface tension, density, the pH, water activity refractive index, and total soluble solids (TSS) of honey and GFSw/.

Sample	Surface Tension, mN/m	Density, g/cm^3^	pH	Water Activity	Refractive Index	TSS, ^0^Brix
Honey	80.292 ± 0.167	1.4207 ± 0.00025	3.75 ± 0.045	0.586 ± 0.00059	1.490 ± 0.00075	80.075 ± 0.09
GFSw	82.214 ± 0.015	1.4301± 0.00092	4.31 ± 0.032	0.555 ± 0.00134	1.495 ± 0.000	82.025 ± 0.15

**Table 7:** Combination index (CI) between samples in AOA assays.

AOA Method	GFSw_RE	GFSw_CA	GFSw_EP
FRAP	0.986 ± 0.021	0.973 ± 0.036	1.149 ± 0.056
CUPRAC	1.409 ± 0.023	1.605 ± 0.065	1.502 ± 0.176
DPPH IC_50_	0.915 ± 0.043	1.642 ± 0.085	1.884 ± 0.097
DPPH IC_20_	0.572 ± 0.071	1.077 ± 0.088	1.337 ± 0.108
ABTS IC_50_	2.646 ± 0.162	1.564 ± 0.078	1.310 ± 0.062
ABTS IC_20_	1.011 ±0.079	0.897 ± 0.036	1.241 ± 0.047

GFSw_RE-mixture of NaDES and raspberry extract, GFSw_CA-mixture of NaDES-caffeic acid, GFSw_EP—mixture of NaDES-epicatehin. IC_50_ and IC_20_ represent the analysis for doses at 50% and 20% substrate inhibition, respectively.

**Table 8:** CI values for AOA activity from Table 3 and Table 7 together.

H		RE		CA		EP	
	GFSw						
**FRAP**		0.929 ± 0.029		0.866 ± 0.021		1.104 ± 0.071	
			0.986 ± 0.021		0.973 ± 0.036		1.149 ± 0.056
**CUPRAC**		1.069 ± 0.059		1.426 ± 0.016		1.641 ± 0.086	
			1.409 ± 0.023		1.605 ± 0.065		1.502 ± 0.176
**DPPH 50%**		0.532 ± 0.003		1.174 ± 0.083		2.885 ± 0.183	
			0.915 ± 0.043		1.642 ± 0.085		1.884 ± 0.097
**ABTS 50%**		2.237 ± 0.043		1.552 ± 0.081		1.292± 0.079	
			2.646 ± 0.162		1.564 ± 0.078		1.310 ± 0.062
**DPPH 20%**		0.604 ± 0.019		0.836 ± 0.030		1.436 ± 0.110	
			0.572 ± 0.071		1.077 ± 0.088		1.337 ± 0.108
**ABTS 20%**		0.731 ± 0.032		0.790 ± 0.026		1.438 ± 0.055	
			1.011 ± 0.079		0.897 ± 0.036		1.241 ± 0.047

**Table 9:** CI value intervals and color codification for AOA activity *.

H		RE		CA		EP	
	GFSw						
**FRAP**		0 (+1)		**+1**		−1	
			0 (+1)		0 (+1)		−1
**CUPRAC**		0 (−1)		−1		−2	
			−1		−2		−2
**DPPH 50%**		+2		−1		−3	
			0 (+1)		−2		−2
**ABTS 50%**		−3		−2		−1	
			−3		−2		−1
**DPPH 20%**		+2		+1		−1	
			+2		0 (−1)		−1
**ABTS 20%**		+1		+1		−1	
			0 (−1)		+1		−1

* The values in brackets indicate the tendency, either towards synergism (+1) or antagonism (−1). The color code indicates highly similar behaviour between honey and GFSw (dark orange) and moderate similar behaviour between honey and GFSw (light orange).

## Data Availability

All the data is contained within the article.

## Supplementary Materials

The following supporting information can be downloaded at: https://www.mdpi.com/article/10.3390/antiox11112194/s1.
