# Supplementary material for: Honey and Its Biomimetic Deep Eutectic Solvent Modulate the Antioxidant Activity of Polyphenols
Source: Antioxidants (Basel). 2022 Nov 6;11(11):2194. doi: 10.3390/antiox11112194 (PMC9686586; doi:10.3390/antiox11112194)
Supplement: Supplementary file 1 [file antioxidants-11-02194-s001.zip › antioxidants-1961785-supplementary.pdf]

Supplementary Material for manuscript:

Honey and its biomimetic deep eutectic solvent modulate the antioxidant activity of polyphenols

by Luminița Dimitriu, Diana Constantinescu-Aruxandei, Daniel Preda, Andra-Lavinia Nichițean, Cristian-Andi Nicolae, Victor Faraon, Marius Ghiurea, Mihaela Ganciarov, Narcisa Elena Băbeanu, and Florin Oancea

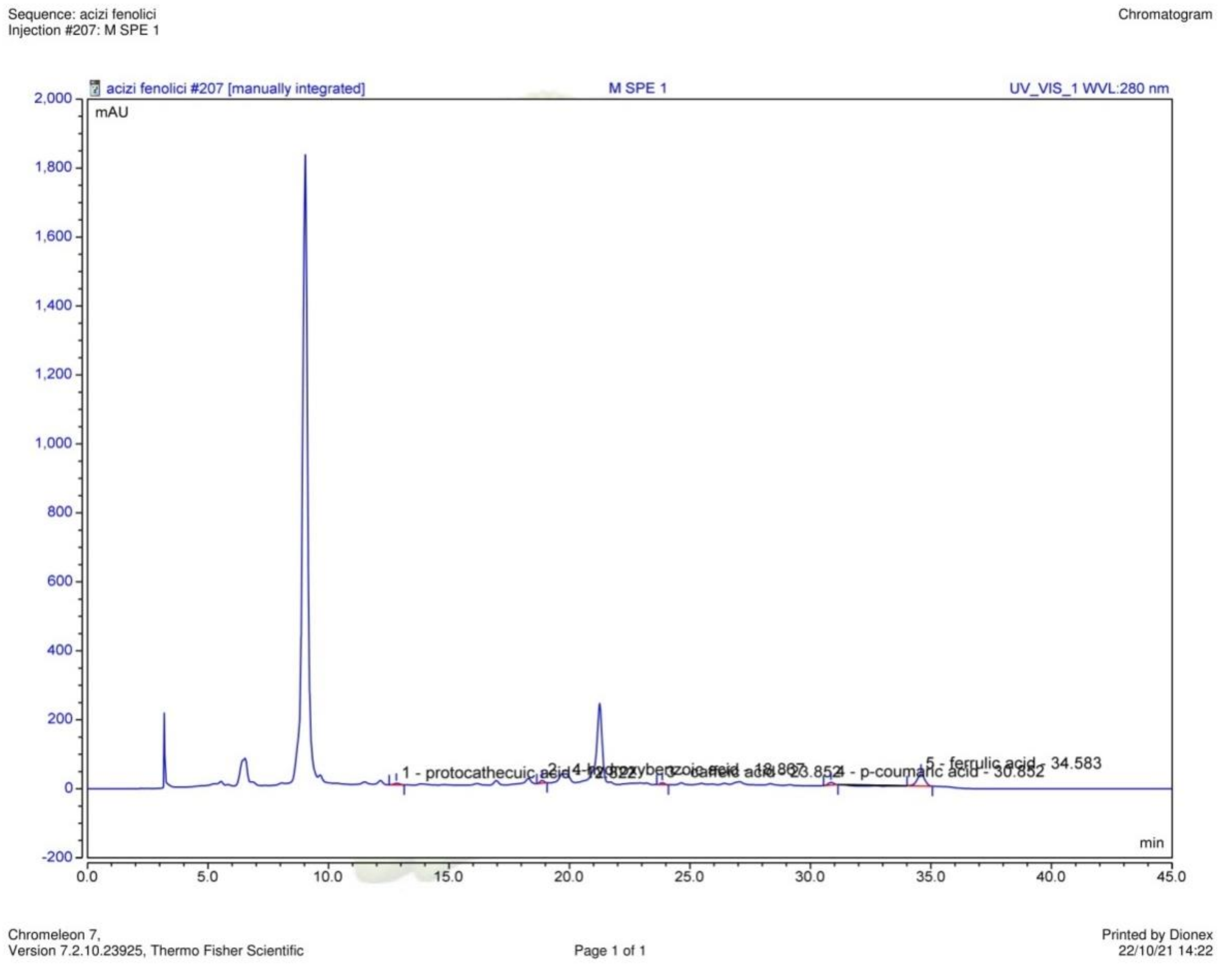

Figure S1. HPLC chromatogram of phenolic acids from honey

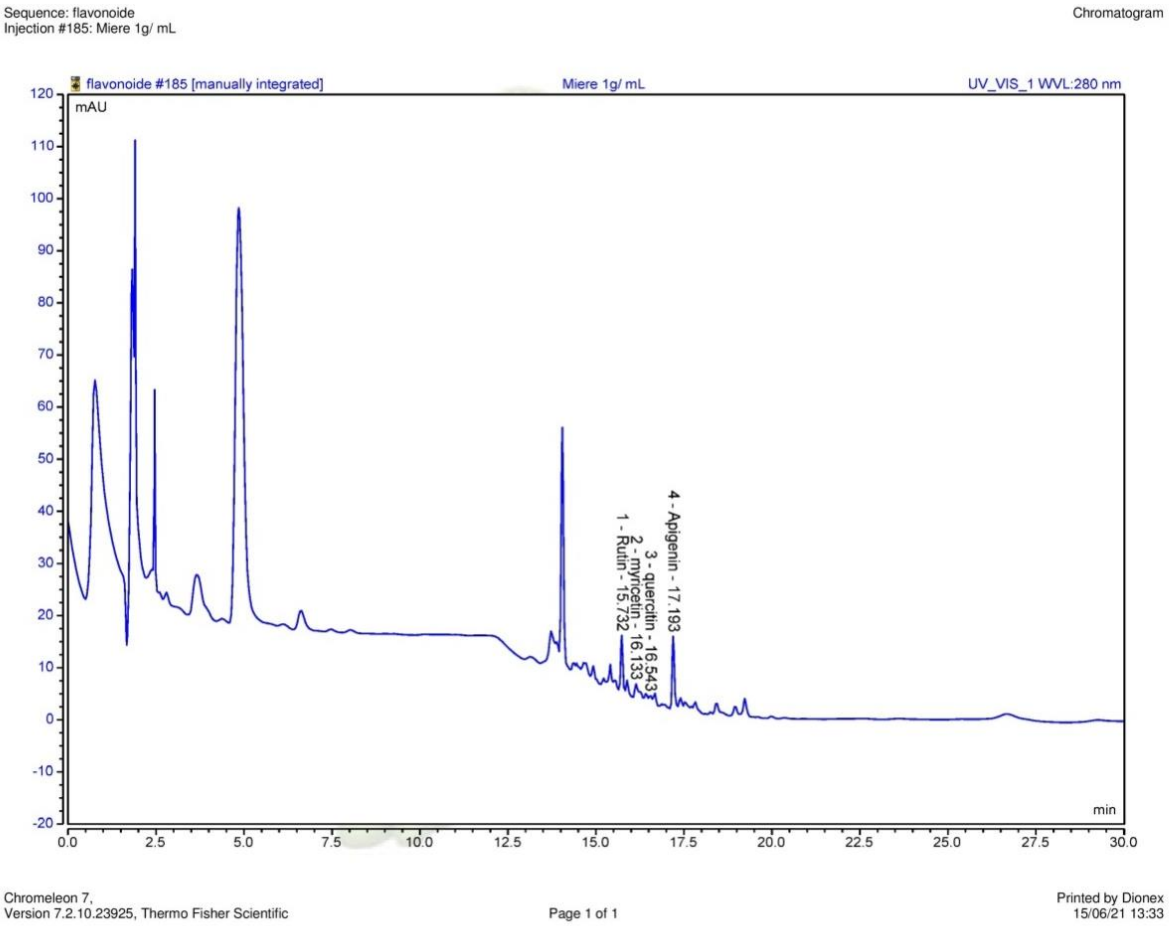

Figure S2. HPLC chromatogram of flavonoids from honey

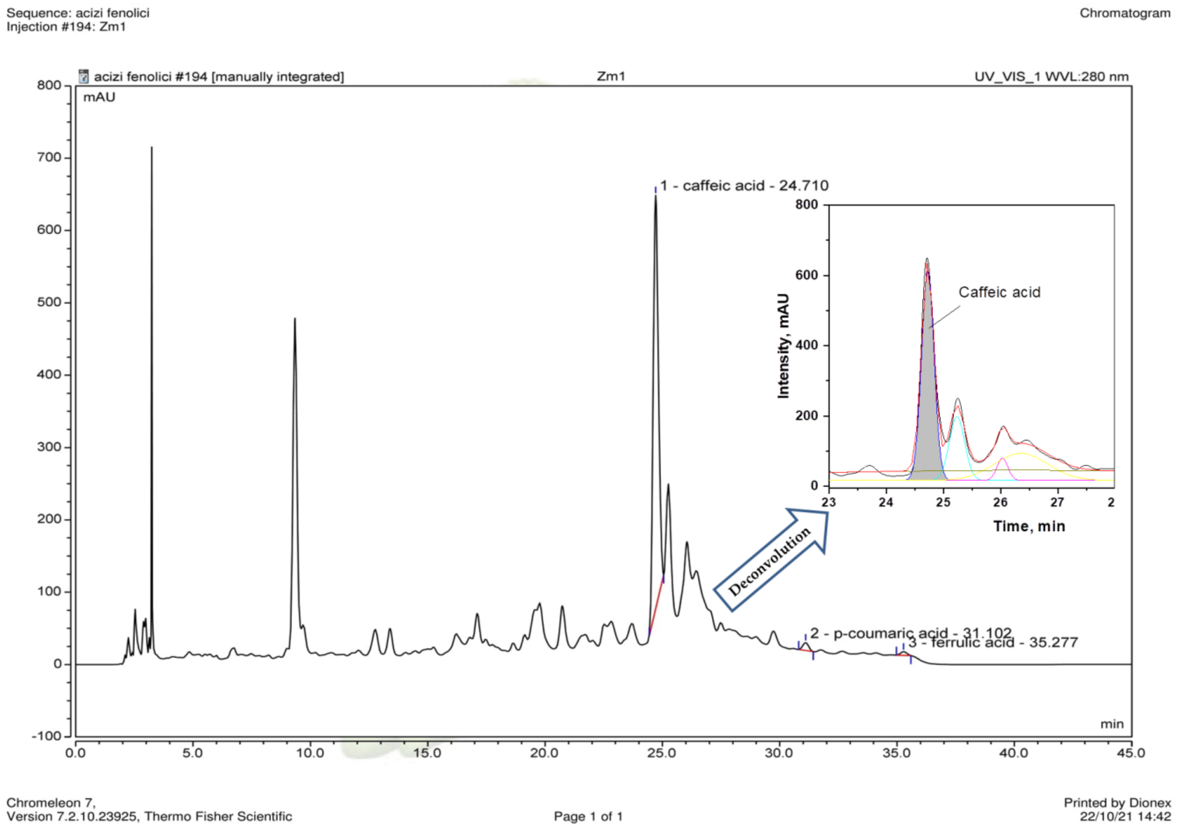

Figure S3. HPLC chromatogram of phenolic acids (the red line was set as baseline in HPLC Chromeleon 7.0 software for quantification, it was not used for Origin baseline) from raspberry extract and peak deconvolution in Origin (insert). The caffeic acid determined was aprox. 838 μg/g substrate (HPLC) and 770 μg/g substrate (Origin)

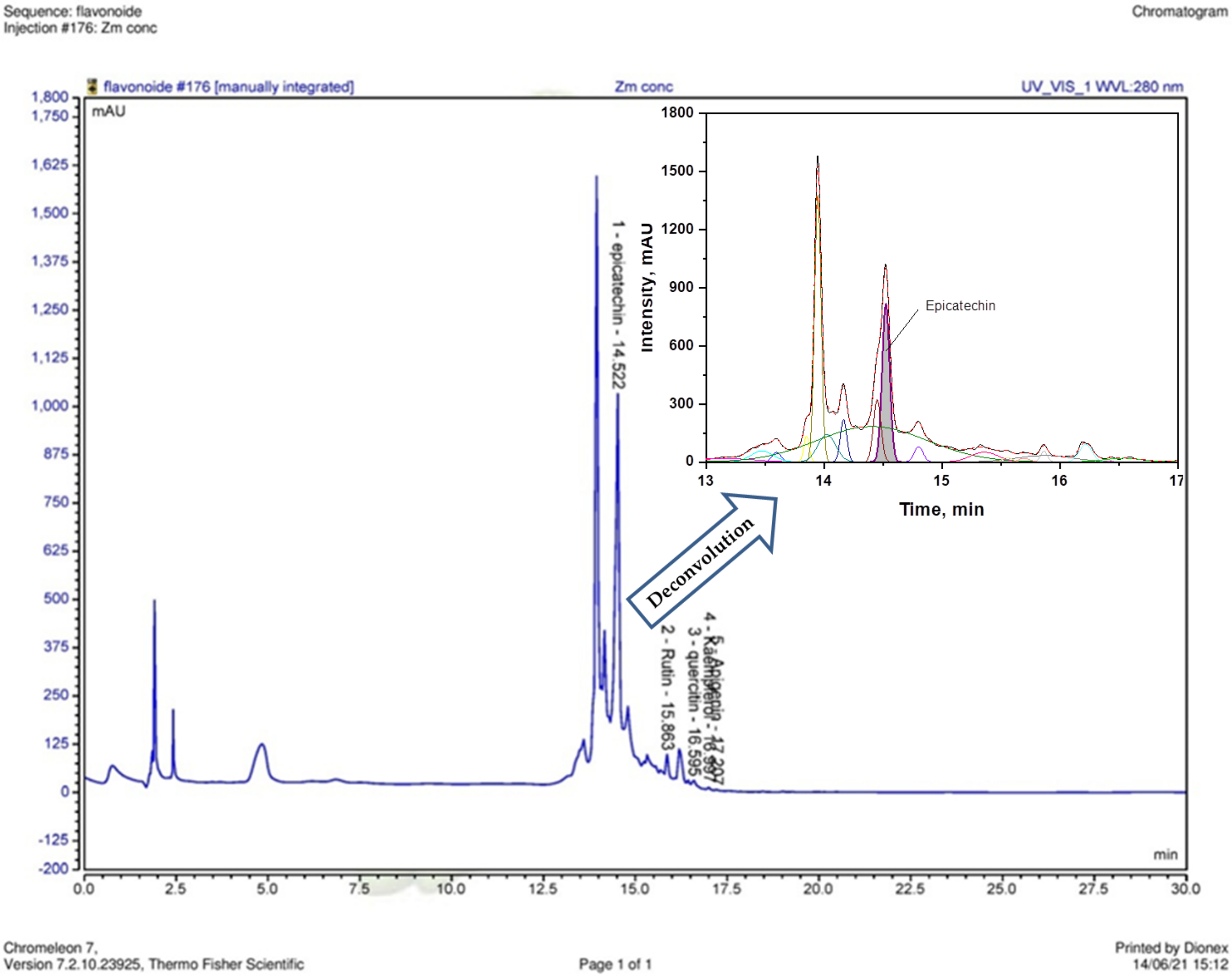

Figure S4. HPLC chromatogram of flavonoids from raspberry extract and peak deconvolution in Origin (insert). The caffeic acid determined was aprox. 2262 µg/g substrate (HPLC) and 1684 µg/g substrate (Origin)

Table S1. Effect-dose curves for the AOA of honey and honey mixtures with raspberry extract, caffeic acid, epicatechin (H_RE, H_CA, and H_EP respectively). The logarithmic fits in the case of DPPH and ABTS have no physical significance, it is just for better visualization.

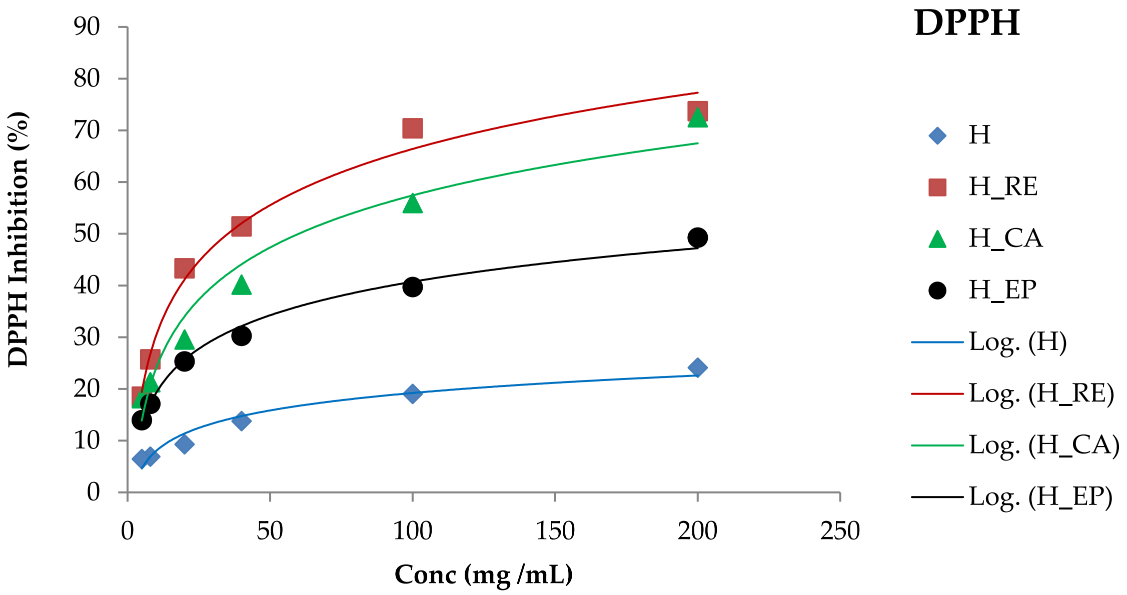

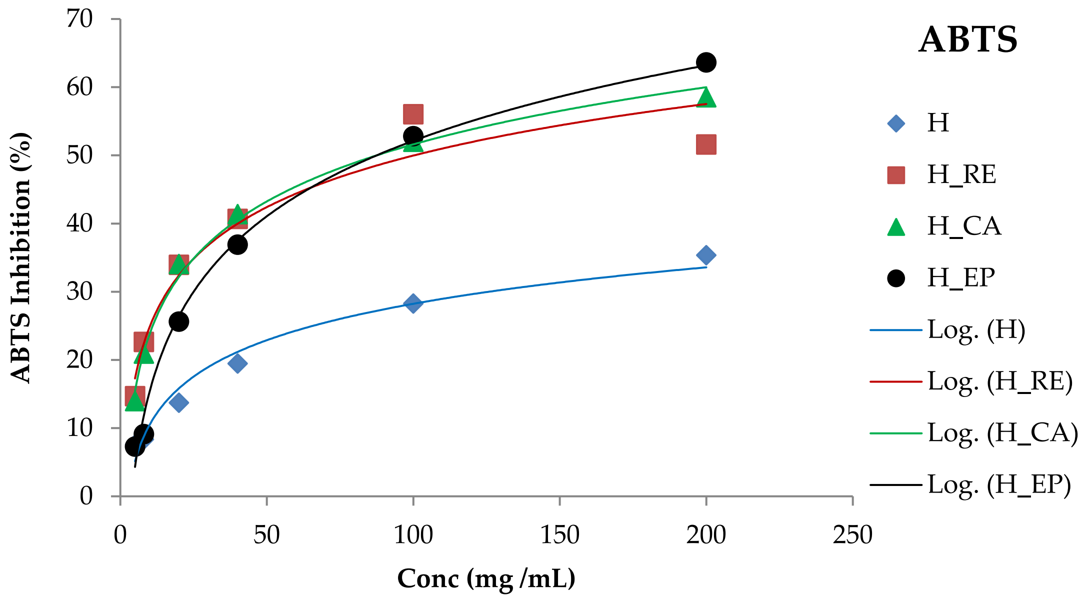

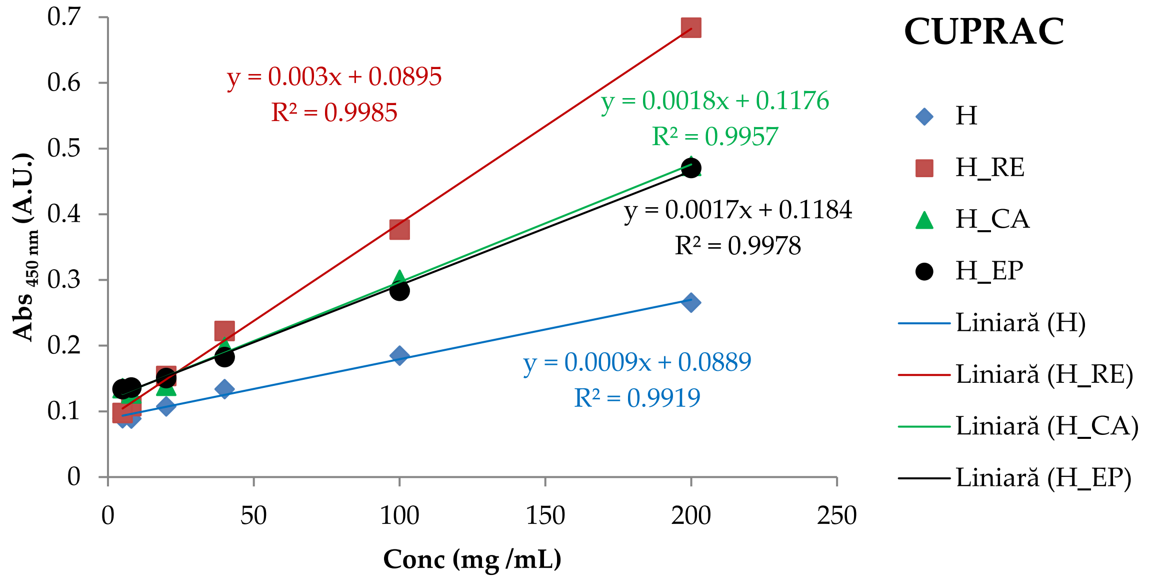
CUPRAC
y = 0.003x + 0.0895
R² = 0.9985
y = 0.0018x + 0.1176
R² = 0.9957
y = 0.0017x + 0.1184
R² = 0.9978
y = 0.0009x + 0.0889
R² = 0.9919
Abs 450 nm (A.U.)
Conc (mg /mL)
H
H_RE
H_CA
H_EP
Liniară (H)
Liniară (H_RE)
Liniară (H_CA)
Liniară (H_EP)

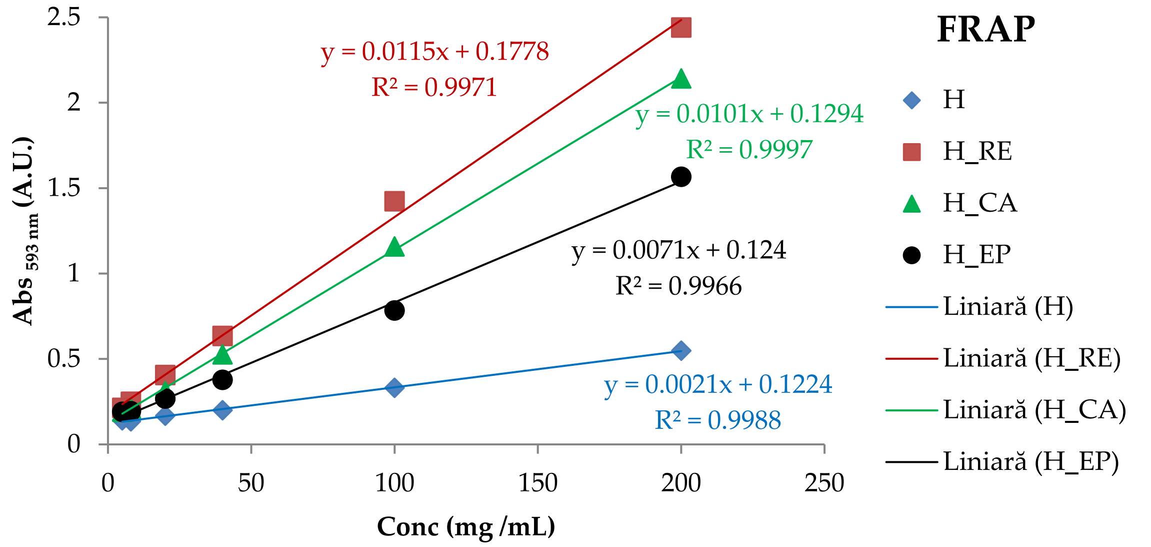
FRAP
y = 0.0115x + 0.1778
R² = 0.9971
y = 0.0101x + 0.1294
R² = 0.9997
y = 0.0071x + 0.124
R² = 0.9966
y = 0.0021x + 0.1224
R² = 0.9988
Abs 593 nm (A.U.)
Conc (mg /mL)
H
H_RE
H_CA
H_EP
Liniară (H)
Liniară (H_RE)
Liniară (H_CA)
Liniară (H_EP)

Table S2. Concentration dependence of experimental and theoretical AOA for H_RE mixture. In the case of FRAP and CUPRAC, the theoretical curve (H+RE) was calculated by absorbance addition. In the case of DPPH and ABTS, the theoretical curve was calculated by Webb analysis (see main text).

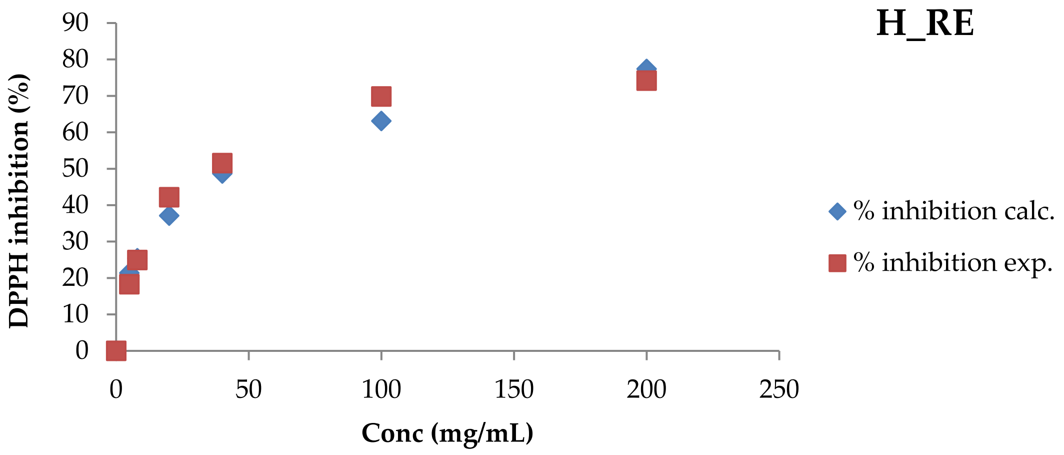

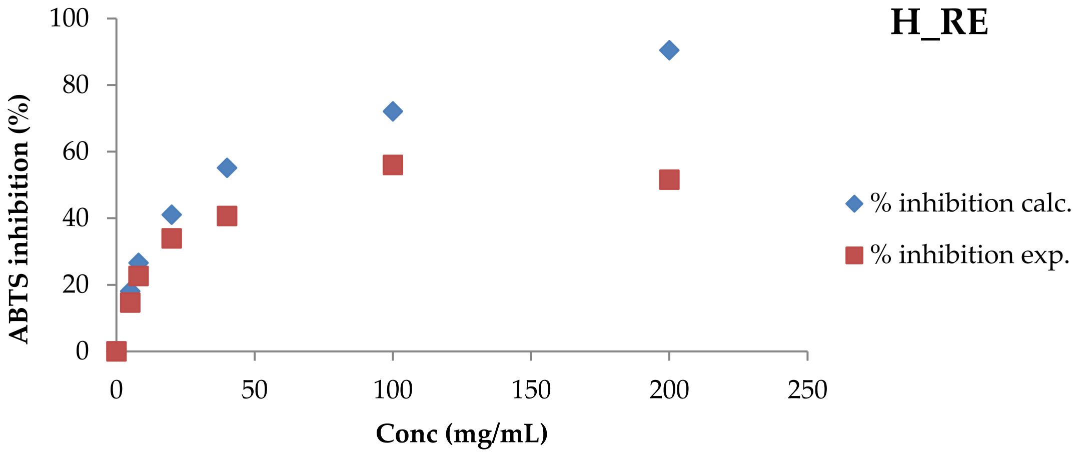

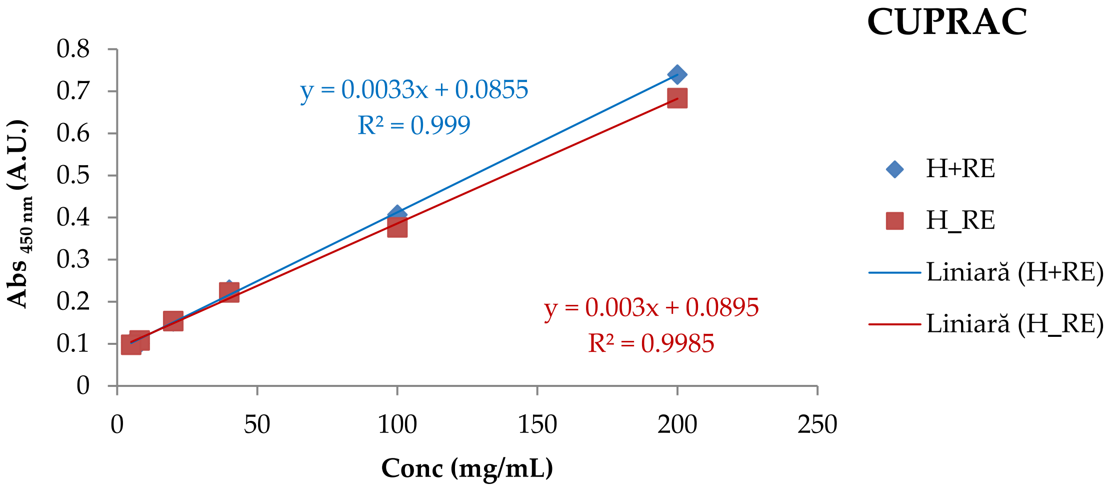

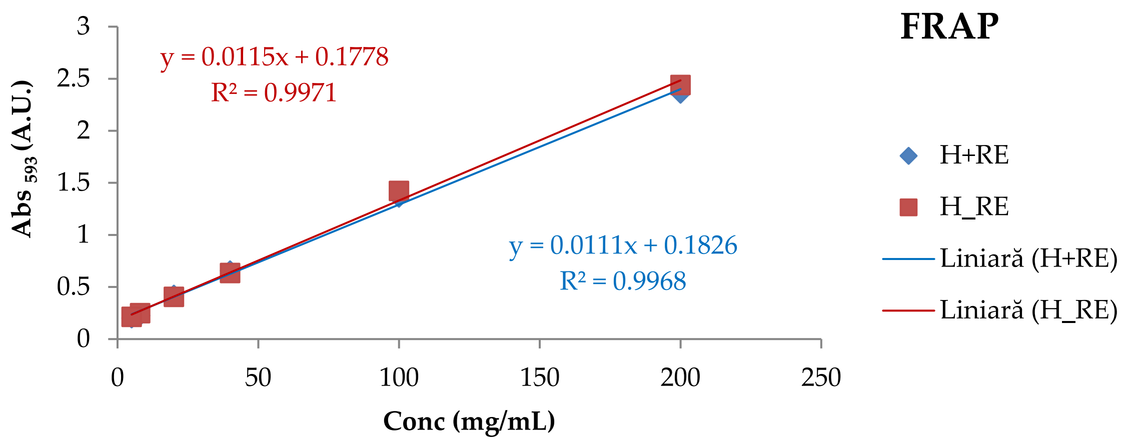

Table S3. Concentration dependence of experimental and theoretical AOA for H_CA and H_EP mixtures. In the case of FRAP and CUPRAC, the theoretical curve (H+CA / H+EP) was calculated by absorbance addition. In the case of DPPH and ABTS, the theoretical curve was calculated by Webb analysis (see main text).

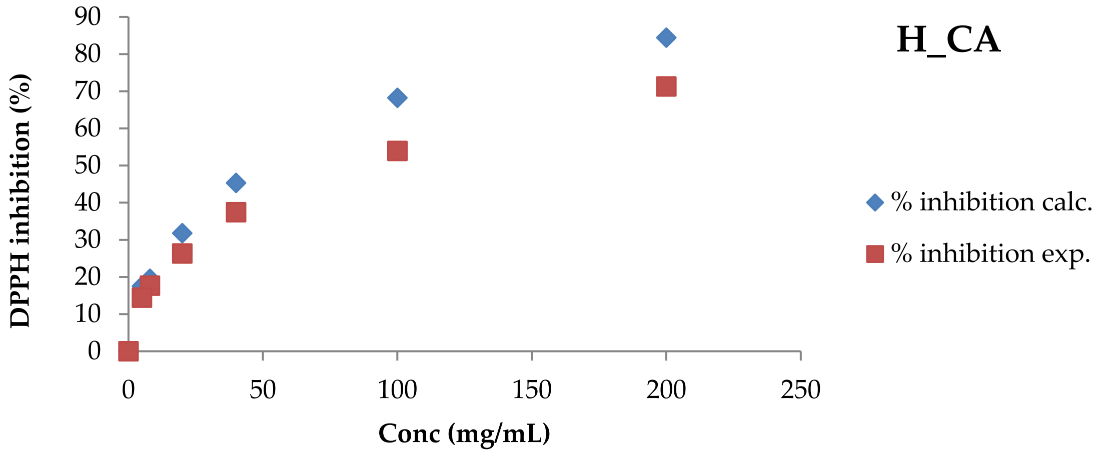

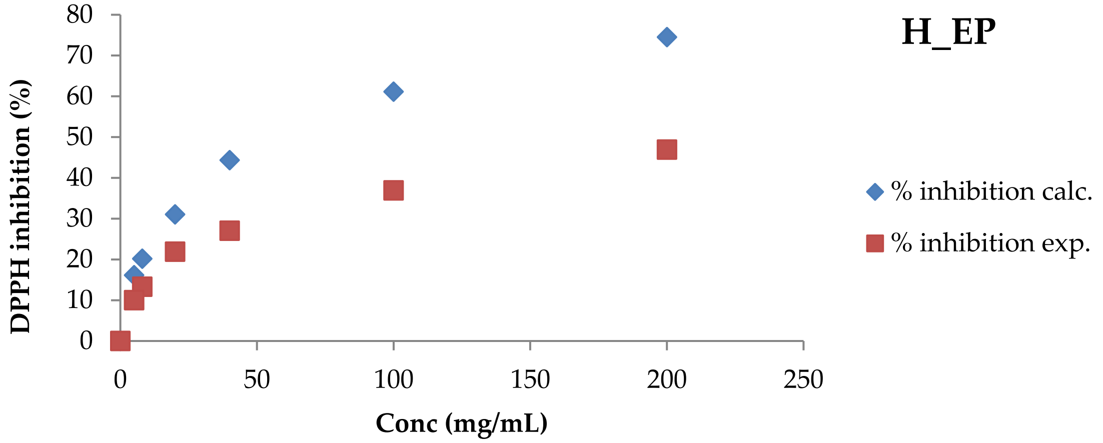

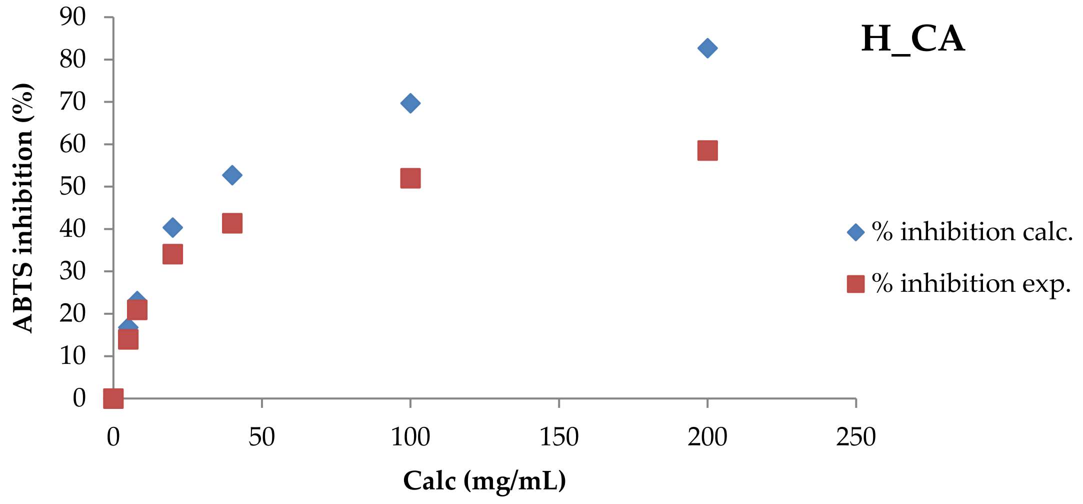
H_CA
ABTS inhibition (%)
Calc (mg/mL)
% inhibition calc.
% inhibition exp.
0
10
20
30
40
50
60
70
80
90
0
50
100
150
200
250

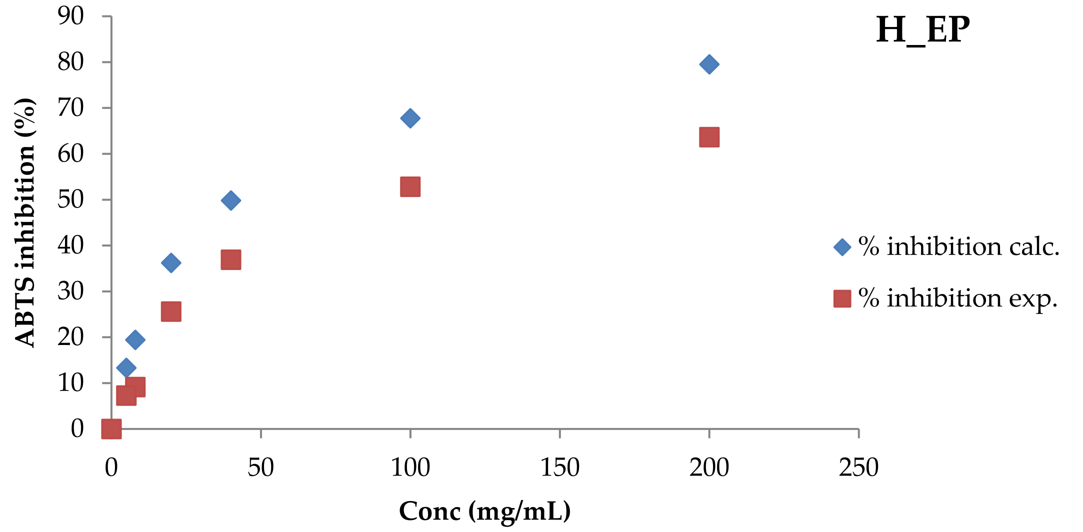
H_EP
ABTS inhibition (%)
Conc (mg/mL)
% inhibition calc.
% inhibition exp.
0
10
20
30
40
50
60
70
80
90
0
50
100
150
200
250

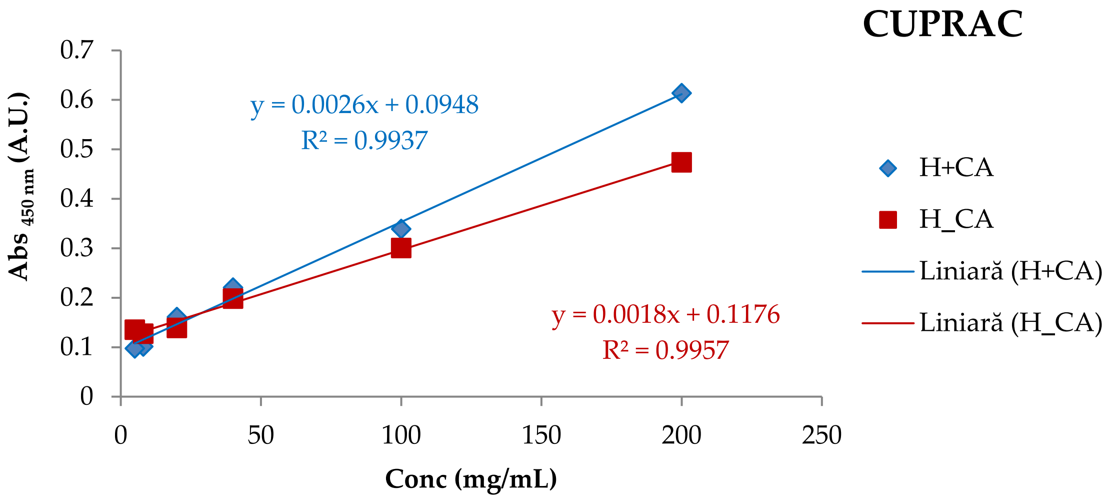
CUPRAC
Abs 450 nm (A.U.)
Conc (mg/mL)
y = 0.0026x + 0.0948
R² = 0.9937
y = 0.0018x + 0.1176
R² = 0.9957
H+CA
H_CA
Liniară (H+CA)
Liniară (H_CA)
0
0.1
0.2
0.3
0.4
0.5
0.6
0.7
0
50
100
150
200
250

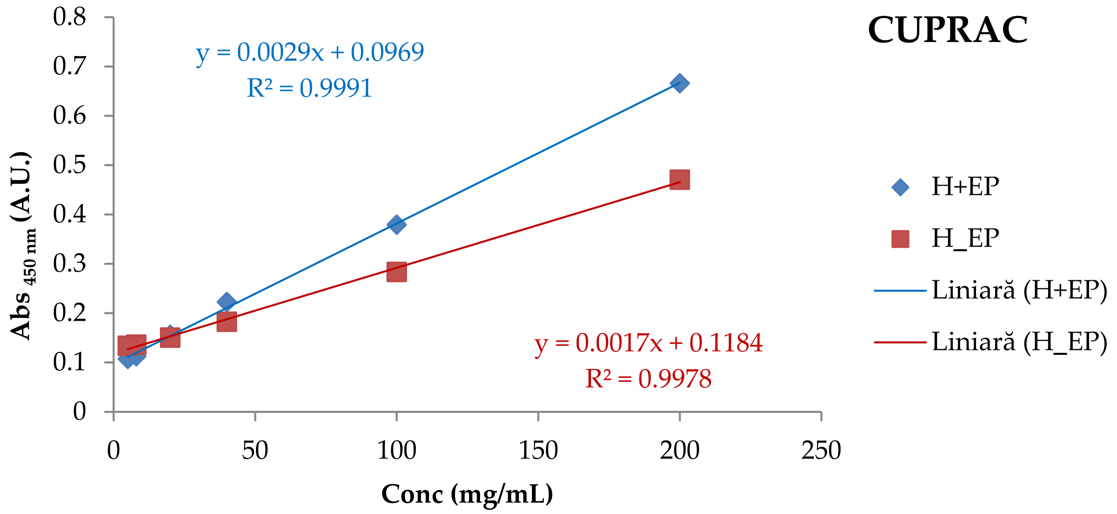
CUPRAC
y = 0.0029x + 0.0969
R² = 0.9991
y = 0.0017x + 0.1184
R² = 0.9978
H+EP
H_EP
Liniară (H+EP)
Liniară (H_EP)
Abs 450 nm (A.U.)
Conc (mg/mL)

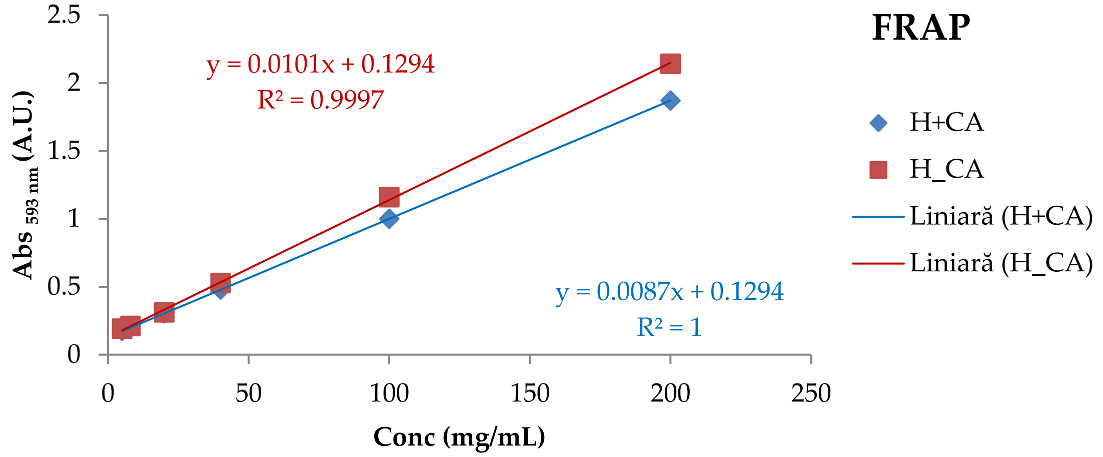
FRAP
y = 0.0101x + 0.1294
R² = 0.9997
y = 0.0087x + 0.1294
R² = 1
H+CA
H_CA
Liniară (H+CA)
Liniară (H_CA)
Abs 593 nm (A.U.)
Conc (mg/mL)

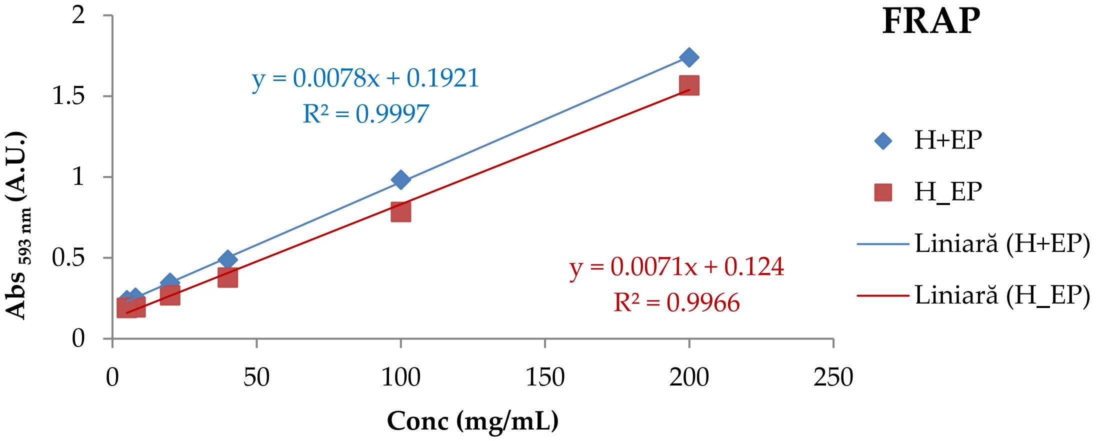
FRAP
y = 0.0078x + 0.1921
R² = 0.9997
y = 0.0071x + 0.124
R² = 0.9966
H+EP
H_EP
Liniară (H+EP)
Liniară (H_EP)
Abs 593 nm (A.U.)
Conc (mg/mL)

Table S4. Effect-dose curves for the AOA of GFSw and GFSw mixtures with raspberry extract, caffeic acid, epicatechin (GFSw_RE, GFSw_CA, and GFSw_EP respectively). The logarithmic fits in the case of DPPH and ABTS have no physical significance, it is just for better visualization.

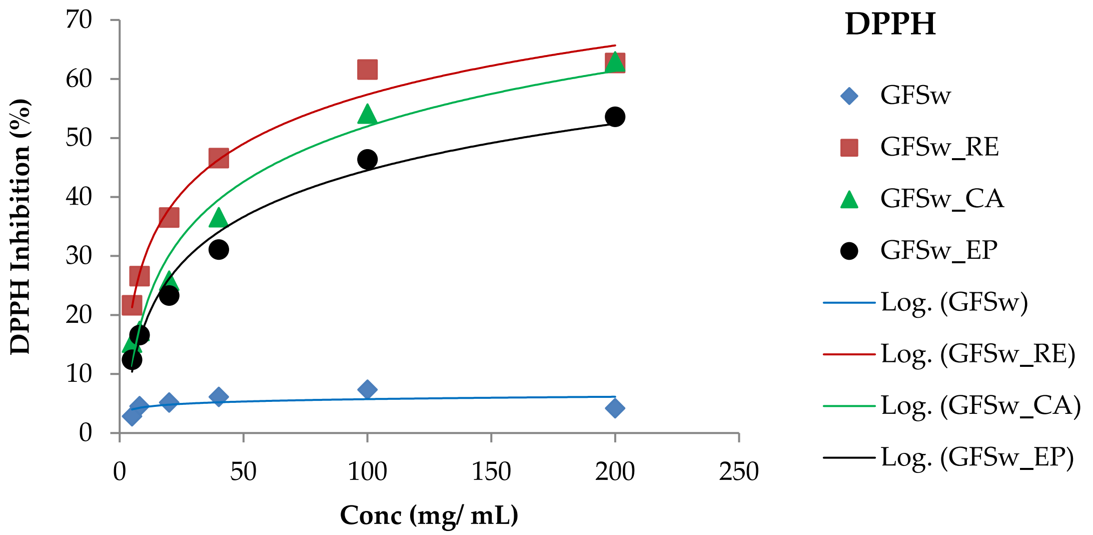

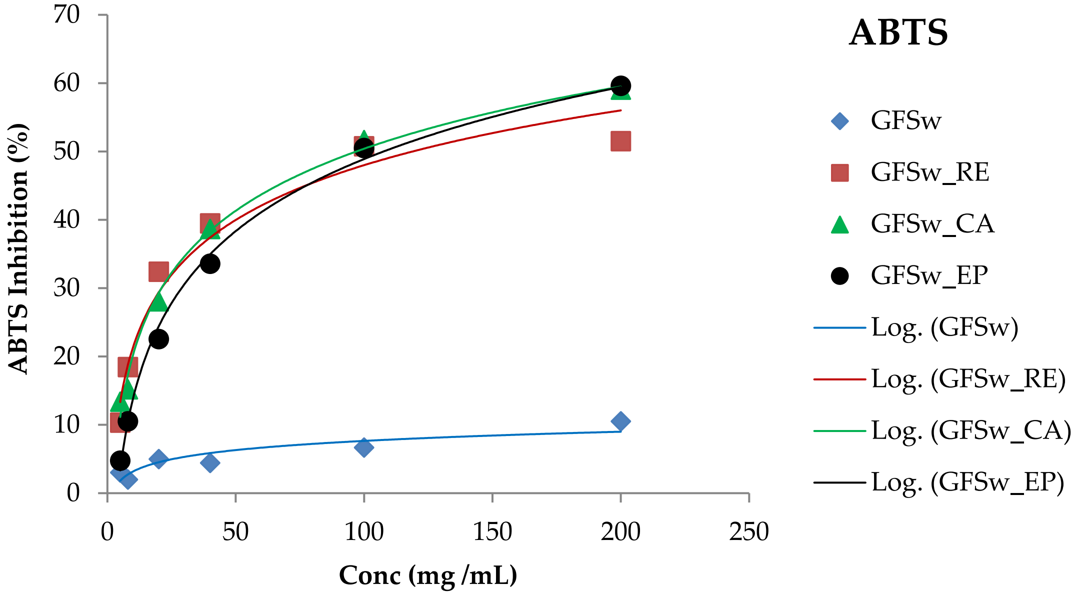

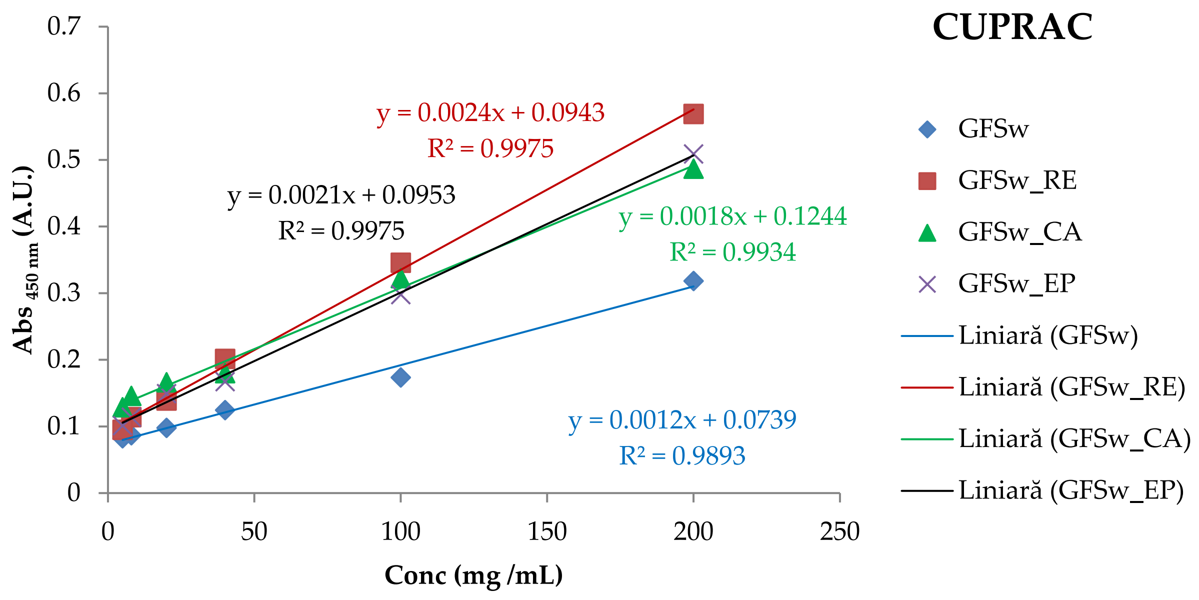

CUPRAC
Abs 450 nm (A.U.)
Conc (mg /mL)
0.7
0.6
0.5
0.4
0.3
0.2
0.1
0
0
50
100
150
200
250
y = 0.0024x + 0.0943
R² = 0.9975
y = 0.0021x + 0.0953
R² = 0.9975
y = 0.0018x + 0.1244
R² = 0.9934
y = 0.0012x + 0.0739
R² = 0.9893
GFSw
GFSw_RE
GFSw_CA
GFSw_EP
Liniară (GFSw)
Liniară (GFSw_RE)
Liniară (GFSw_CA)
Liniară (GFSw_EP)

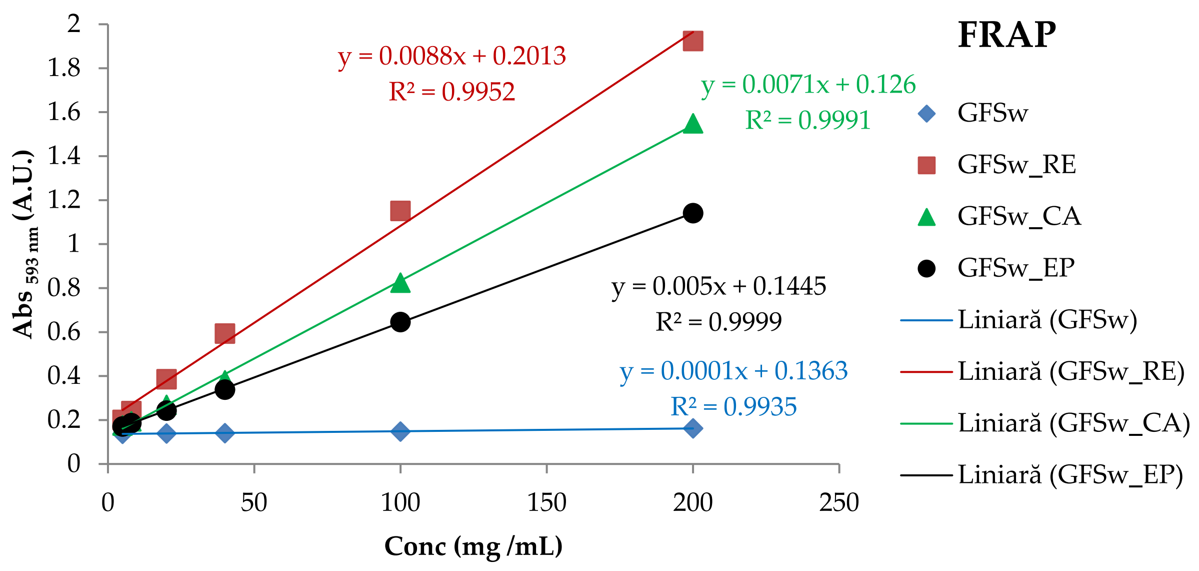

FRAP
Abs 593 nm (A.U.)
Conc (mg /mL)
2
1.8
1.6
1.4
1.2
1
0.8
0.6
0.4
0.2
0
0
50
100
150
200
250
y = 0.0088x + 0.2013
R² = 0.9952
y = 0.0071x + 0.126
R² = 0.9991
y = 0.005x + 0.1445
R² = 0.9999
y = 0.0001x + 0.1363
R² = 0.9935
GFSw
GFSw_RE
GFSw_CA
GFSw_EP
Liniară (GFSw)
Liniară (GFSw_RE)
Liniară (GFSw_CA)
Liniară (GFSw_EP)

Table S5. Concentration dependence of experimental and theoretical AOA for GFSw_RE, GFSw_CA and GFSw_EP mixtures. In the case of FRAP and CUPRAC, the theoretical curve (H+RE / H+CA / H+EP) was calculated by absorbance addition. In the case of DPPH and ABTS, the theoretical curve was calculated by Webb analysis (see main text).

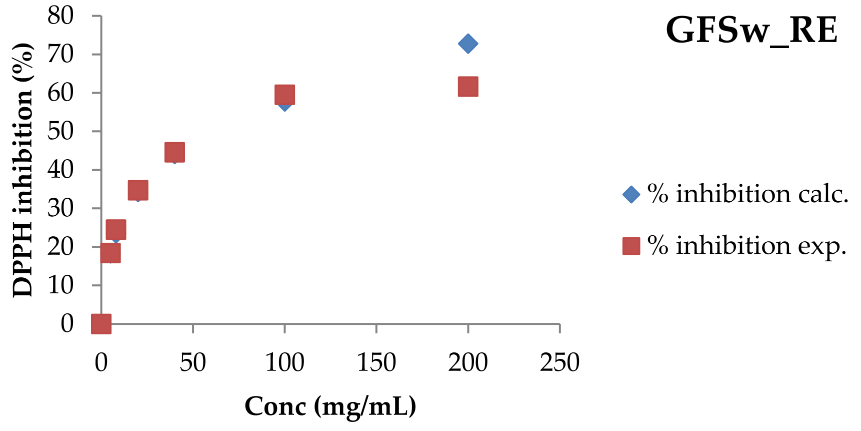

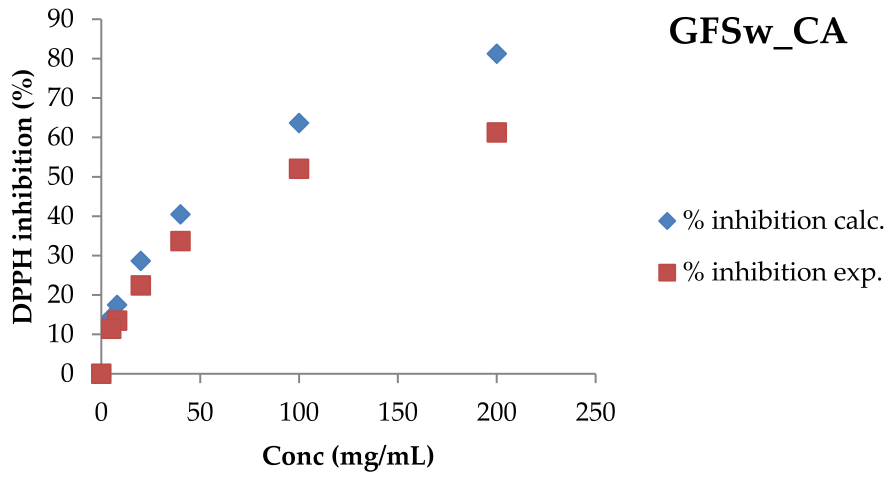

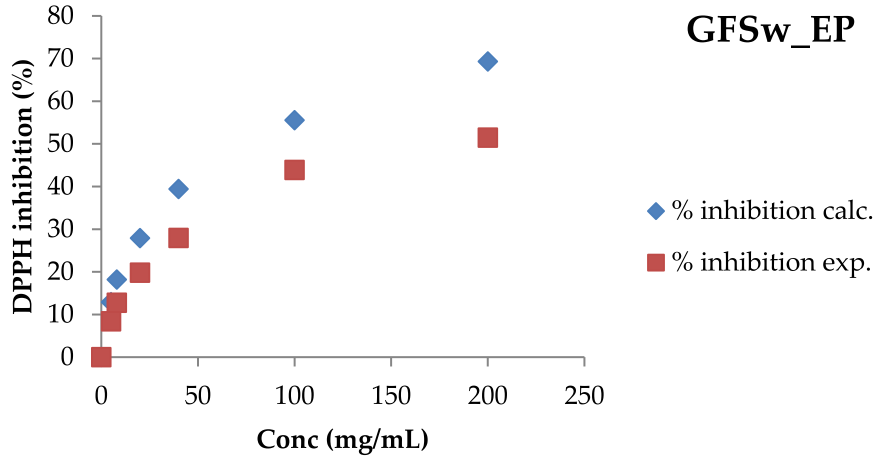

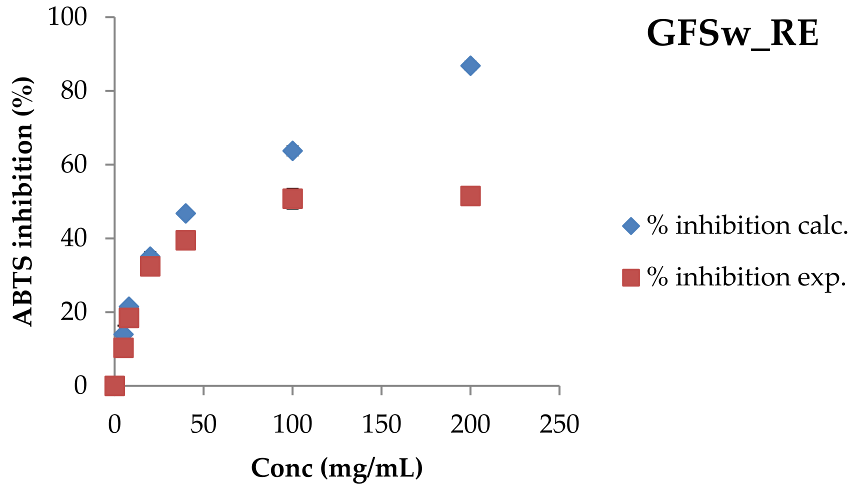
GFSw_RE
ABTS inhibition (%)
100
80
60
40
20
0
0
50
100
150
200
250
Conc (mg/mL)
% inhibition calc.
% inhibition exp.

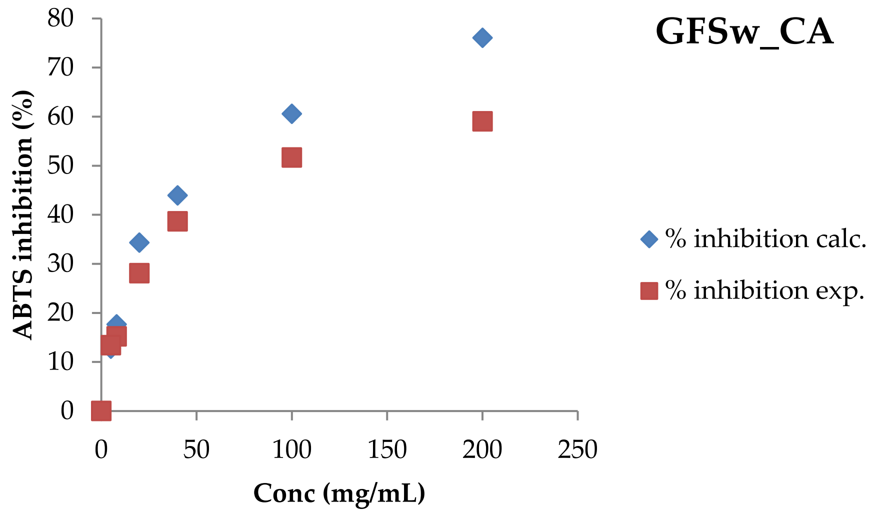
GFSw_CA
ABTS inhibition (%)
80
70
60
50
40
30
20
10
0
0
50
100
150
200
250
Conc (mg/mL)
% inhibition calc.
% inhibition exp.

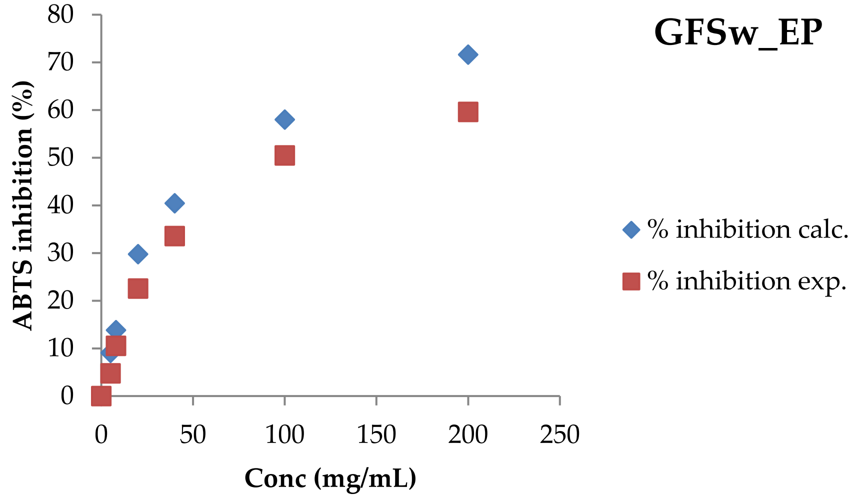
GFSw_EP
ABTS inhibition (%)
80
70
60
50
40
30
20
10
0
0
50
100
150
200
250
Conc (mg/mL)
% inhibition calc.
% inhibition exp.

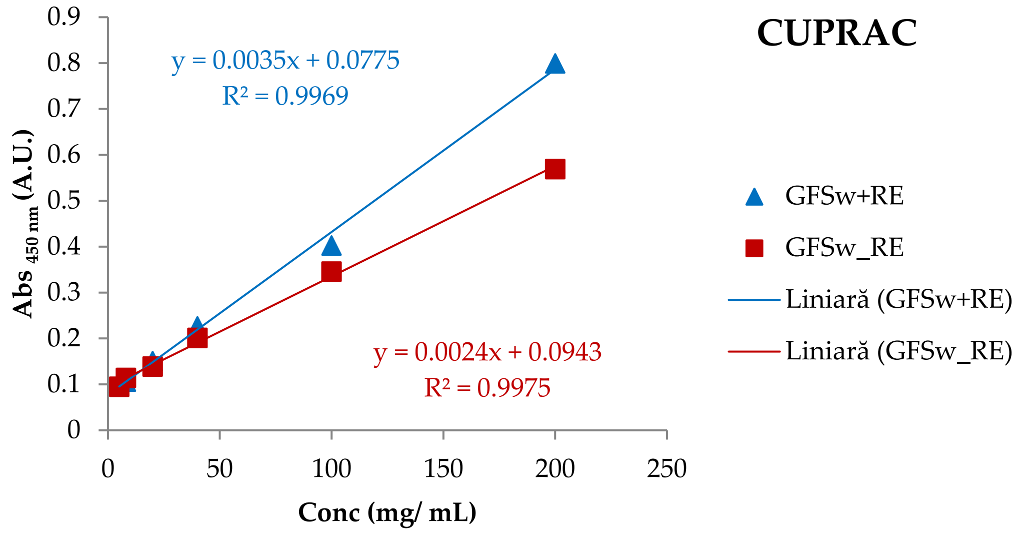
CUPRAC
y = 0.0035x + 0.0775
R² = 0.9969
y = 0.0024x + 0.0943
R² = 0.9975
Abs 450 nm (A.U.)
Conc (mg/ mL)
GFSw+RE
GFSw_RE
Liniară (GFSw+RE)
Liniară (GFSw_RE)

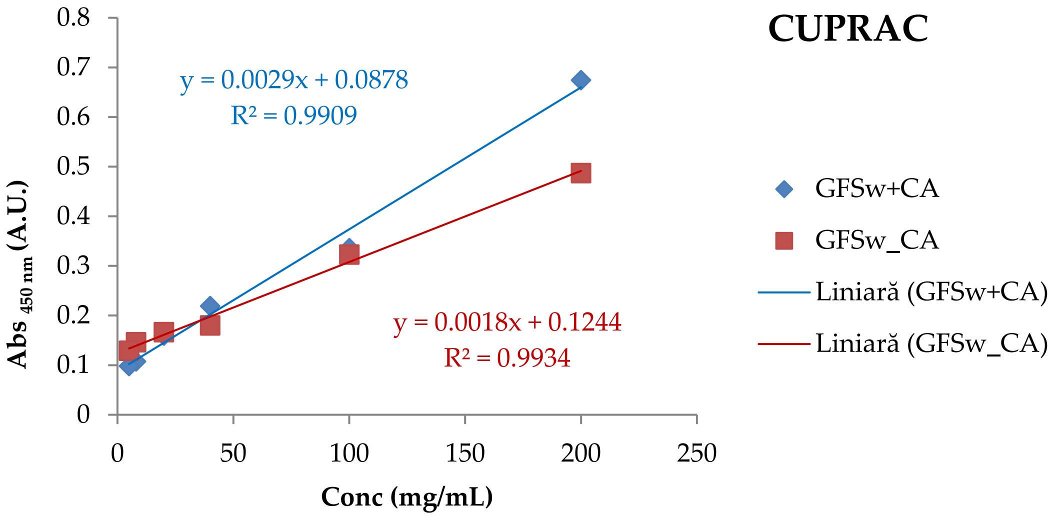
CUPRAC
y = 0.0029x + 0.0878
R² = 0.9909
y = 0.0018x + 0.1244
R² = 0.9934
Abs 450 nm (A.U.)
Conc (mg/mL)
GFSw+CA
GFSw_CA
Liniară (GFSw+CA)
Liniară (GFSw_CA)

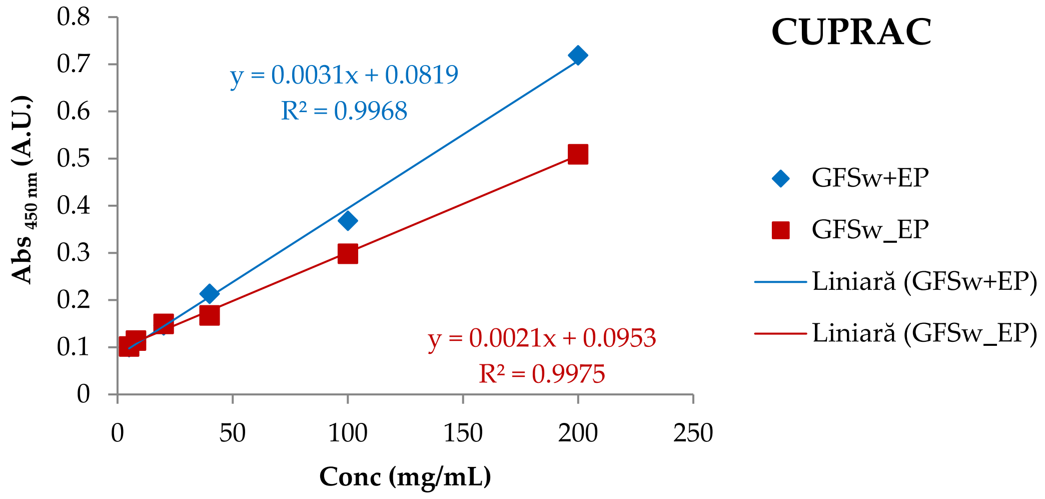
CUPRAC
y = 0.0031x + 0.0819
R² = 0.9968
y = 0.0021x + 0.0953
R² = 0.9975
Abs 450 nm (A.U.)
Conc (mg/mL)
GFSw+EP
GFSw_EP
Liniară (GFSw+EP)
Liniară (GFSw_EP)

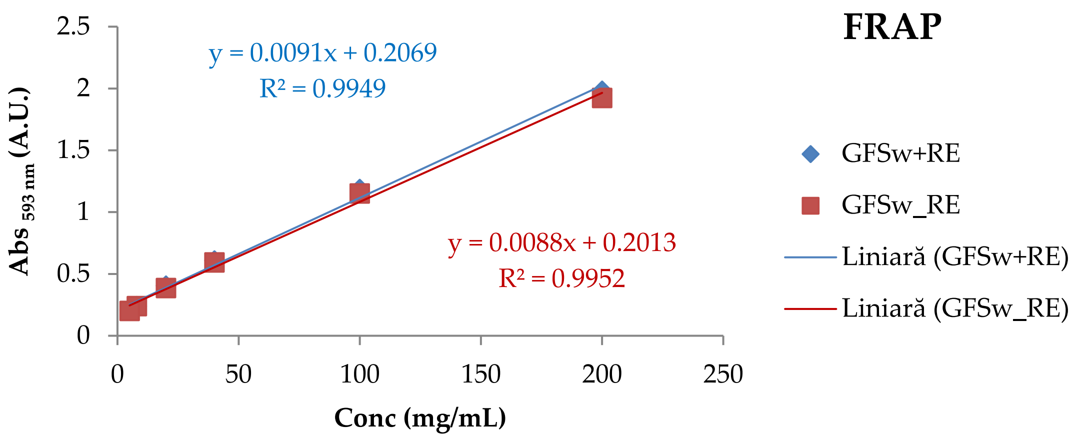
FRAP
2.5
2
1.5
1
0.5
0
Abs 593 nm (A.U.)
y = 0.0091x + 0.2069
R² = 0.9949
y = 0.0088x + 0.2013
R² = 0.9952
0
50
100
150
200
250
Conc (mg/mL)
GFSw+RE
GFSw_RE
Liniară (GFSw+RE)
Liniară (GFSw_RE)

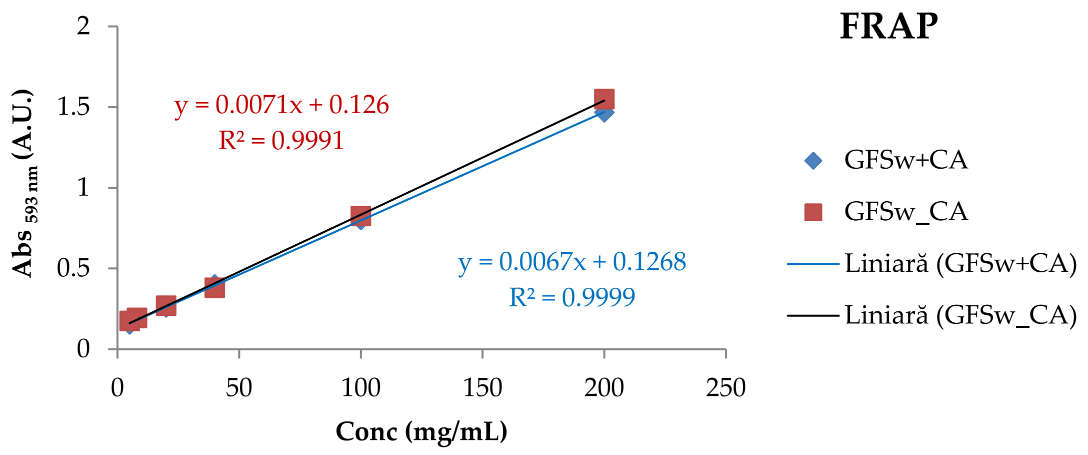
FRAP
2
1.5
1
0.5
0
Abs 593 nm (A.U.)
y = 0.0071x + 0.126
R² = 0.9991
y = 0.0067x + 0.1268
R² = 0.9999
0
50
100
150
200
250
Conc (mg/mL)
GFSw+CA
GFSw_CA
Liniară (GFSw+CA)
Liniară (GFSw_CA)

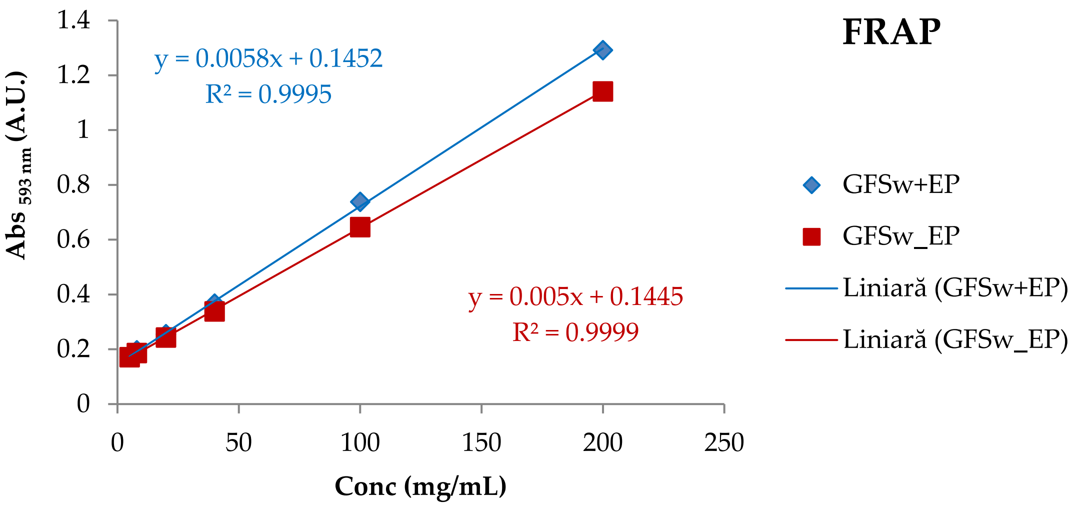
FRAP
1.4
1.2
1
0.8
0.6
0.4
0.2
0
Abs 593 nm (A.U.)
y = 0.0058x + 0.1452
R² = 0.9995
y = 0.005x + 0.1445
R² = 0.9999
0
50
100
150
200
250
Conc (mg/mL)
GFSw+EP
GFSw_EP
Liniară (GFSw+EP)
Liniară (GFSw_EP)

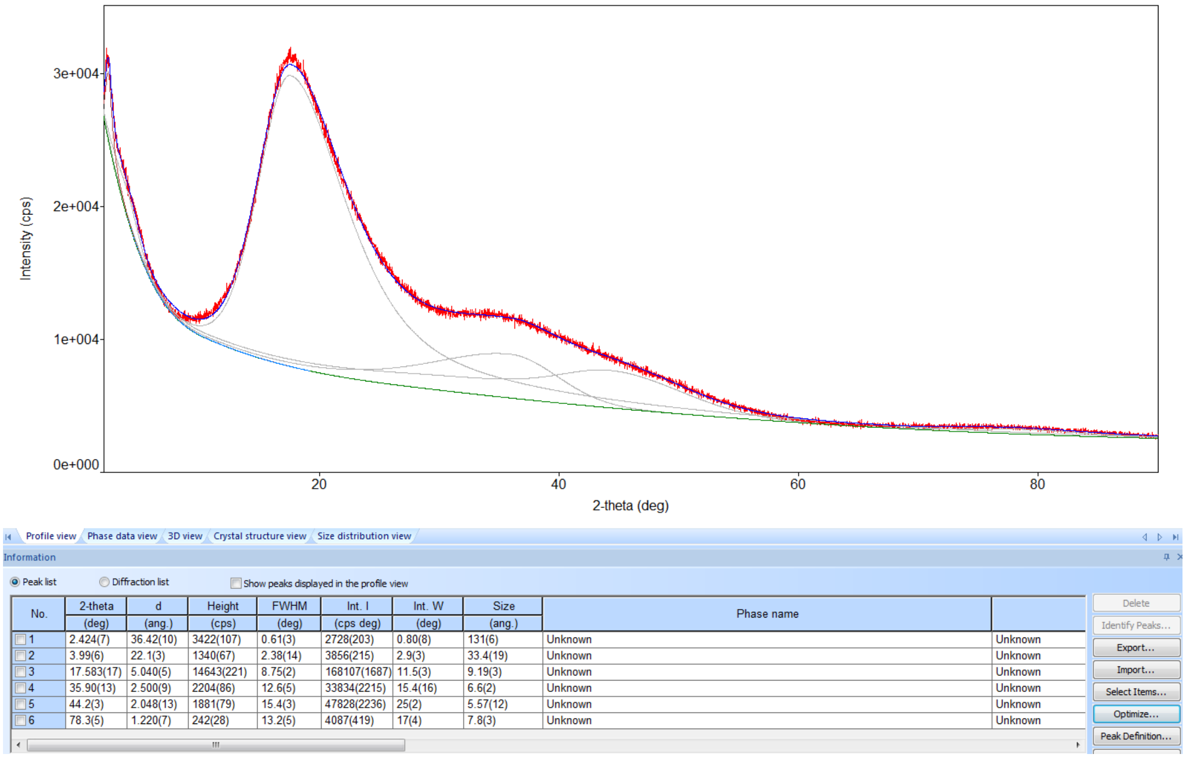

No.	2-theta (deg)	d (ang.)	Height (cps)	FWHM (deg)	Int. I (cps deg)	Int. W (deg)	Size (ang.)	Phase name	
1	2.424(7)	36.42(10)	3422(107)	0.61(3)	2728(203)	0.80(8)	131(6)	Unknown	Unknown
2	3.99(6)	22.1(3)	1340(67)	2.38(14)	3856(215)	2.9(3)	33.4(19)	Unknown	Unknown
3	17.583(17)	5.040(5)	14643(221)	8.75(2)	168107(1687)	11.5(3)	9.19(3)	Unknown	Unknown
4	35.90(13)	2.500(9)	2204(86)	12.6(5)	33834(2215)	15.4(16)	6.6(2)	Unknown	Unknown
5	44.2(3)	2.048(13)	1881(79)	15.4(3)	47828(2236)	25(2)	5.57(12)	Unknown	Unknown
6	78.3(5)	1.220(7)	242(28)	13.2(5)	4087(419)	17(4)	7.8(3)	Unknown	Unknown

Figure S5. Peak deconvolution (4 peaks) of the XRD diffractogram of honey

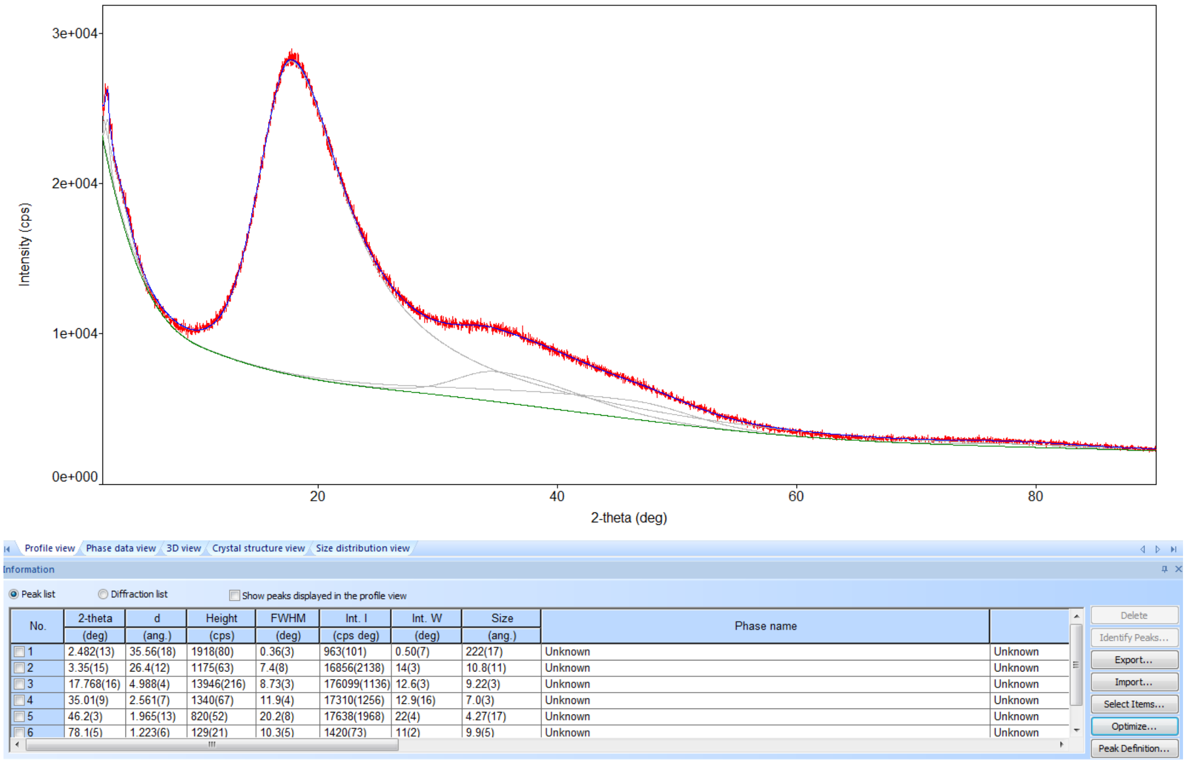

No.	2-theta (deg)	d (ang.)	Height (cps)	FWHM (deg)	Int. I (cps deg)	Int. W (deg)	Size (ang.)	Phase name	
1	2.482(13)	35.56(18)	1918(80)	0.36(3)	963(101)	0.50(7)	222(17)	Unknown	Unknown
2	3.35(15)	26.4(12)	1175(63)	7.4(8)	16856(2138)	14(3)	10.8(11)	Unknown	Unknown
3	17.768(16)	4.988(4)	13946(216)	8.73(3)	176099(1136)	12.6(3)	9.22(3)	Unknown	Unknown
4	35.01(9)	2.561(7)	1340(67)	11.9(4)	17310(1256)	12.9(16)	7.0(3)	Unknown	Unknown
5	46.2(3)	1.965(13)	820(52)	20.2(8)	17638(1968)	22(4)	4.27(17)	Unknown	Unknown
6	78.1(5)	1.223(6)	129(21)	10.3(5)	1420(73)	11(2)	9.9(5)	Unknown	Unknown

Figure S6. Peak deconvolution (4 peaks) of the XRD diffractogram of GFSw

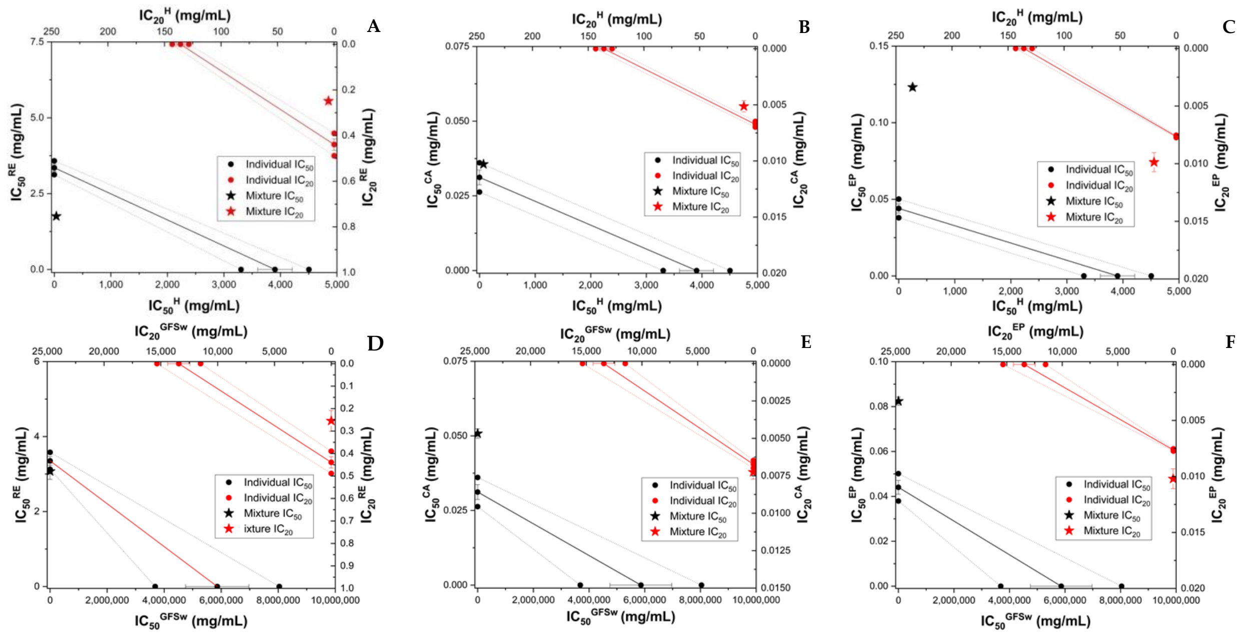

Figure S7. Isobolograms based on IC_{50} (half-maximal inhibitory concentration, black) and IC_{20} (inhibitory concentration at 20% substrate inhibition, red) by DPPH method for H_RE (A) , H_CA (B), H_EP (C), GFSw_RE (D), GFSw_CA (E), and GFSw_EP (F). The error bars from 3 measurements are shown for each value. Confidence intervals at 95% confidence are shown by dashed lines.

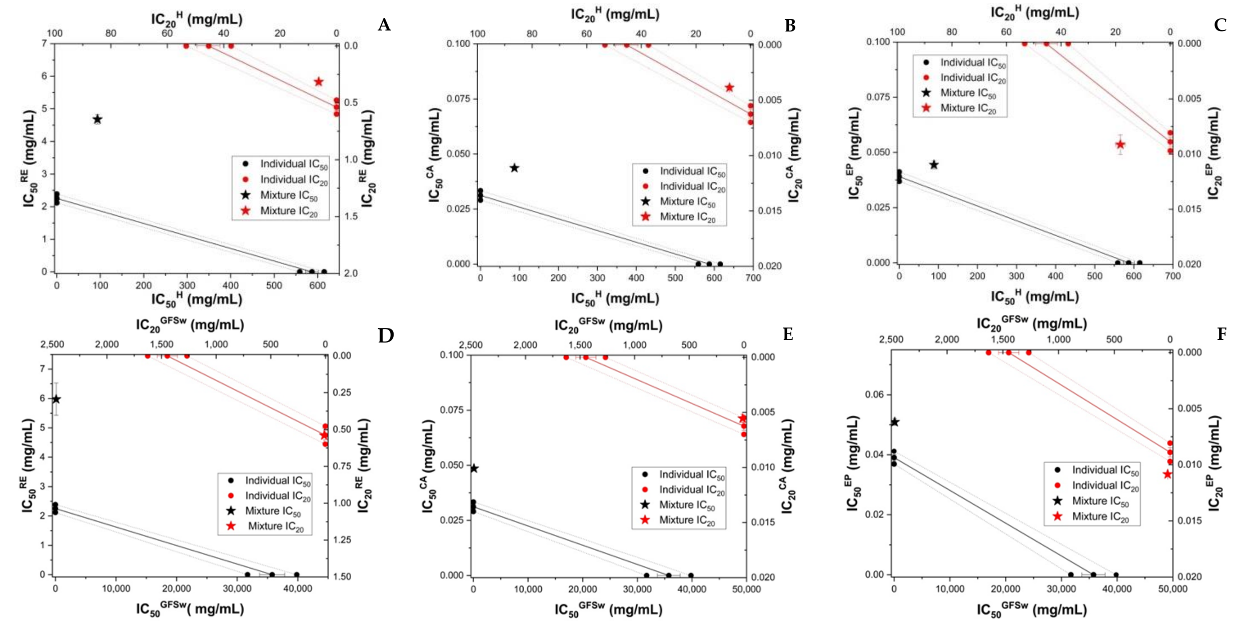

Figure S8. Isobolograms based on IC_{50} (half-maximal inhibitory concentration, black) and IC_{20} (inhibitory concentration at 20% substrate inhibition, red) by ABTS method for H_RE (A) , H_CA (B), H_EP (C), GFSw_RE (D), GFSw_CA (E), and GFSw_EP (F). The error bars from 3 measurements are shown for each value. Confidence intervals at 95% confidence are shown by dashed lines.

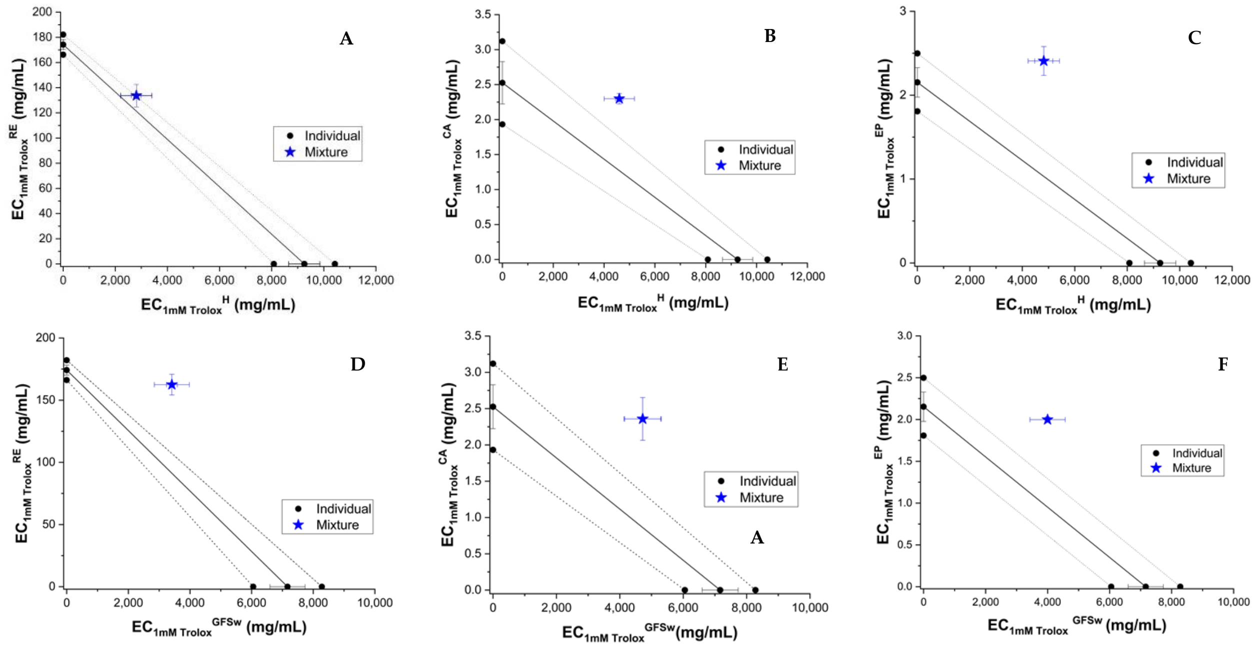

Figure S9. Isobolograms based on 1 mM Trolox equivalent by CUPRAC method for H_RE (A) , H_CA (B), H_EP (C), GFSw_RE (D), GFSw_CA (E), and GFSw_EP (F). The error bars from 3 measurements are shown for each value. Confidence intervals at 95% confidence are shown by dashed lines.

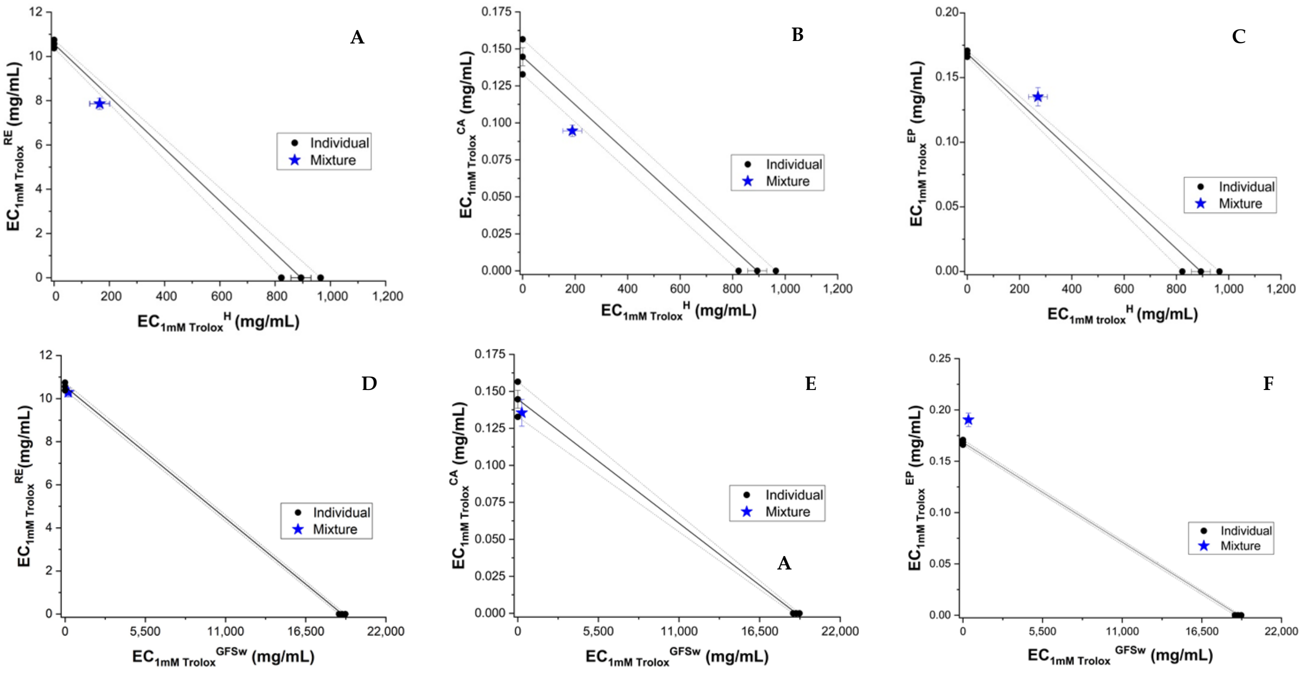

Figure S10. Isobolograms based on 1 mM Trolox equivalent by FRAP method for H_RE (A) , H_CA (B), H_EP (C), GFSw_RE (D), GFSw_CA (E), and GFSw_EP (F). The error bars from 3 measurements are shown for each value. Confidence intervals at 95% confidence are shown by dashed lines.
